# A Network of Multi-Tasking Proteins at the DNA Replication Fork Preserves Genome Stability 

**DOI:** 10.1371/journal.pgen.0010061

**Published:** 2005-12-02

**Authors:** Martin E Budd, Amy Hin Yan Tong, Piotr Polaczek, Xiao Peng, Charles Boone, Judith L Campbell

**Affiliations:** 1 Braun Laboratories, California Institute of Technology, Pasadena, California, United States of America; 2 Banting and Best Department of Medical Research, University of Toronto, Toronto, Ontario, Canada; 3 Department of Medical Genetics and Microbiology, University of Toronto, Toronto, Ontario, Canada; Yale University, United States of America

## Abstract

To elucidate the network that maintains high fidelity genome replication, we have introduced two conditional mutant alleles of *DNA2,* an essential DNA replication gene, into each of the approximately 4,700 viable yeast deletion mutants and determined the fitness of the double mutants. Fifty-six *DNA2*-interacting genes were identified. Clustering analysis of genomic synthetic lethality profiles of each of 43 of the *DNA2*-interacting genes defines a network (consisting of 322 genes and 876 interactions) whose topology provides clues as to how replication proteins coordinate regulation and repair to protect genome integrity. The results also shed new light on the functions of the query gene *DNA2,* which, despite many years of study, remain controversial, especially its proposed role in Okazaki fragment processing and the nature of its in vivo substrates. Because of the multifunctional nature of virtually all proteins at the replication fork, the meaning of any single genetic interaction is inherently ambiguous. The multiplexing nature of the current studies, however, combined with follow-up supporting experiments, reveals most if not all of the unique pathways requiring Dna2p. These include not only Okazaki fragment processing and DNA repair but also chromatin dynamics.

## Synopsis

Maintenance of genome stability from generation to generation is a primary defense against mutation and ensuing disease. Thus, the cell has evolved complex mechanisms, consisting of redundant, partially overlapping pathways, to protect the fidelity of genome inheritance. Using modern genetic screening techniques that allow one to investigate every gene in yeast that might be involved in these pathways, the researchers have defined a network consisting of 322 genes that together safeguard the DNA replication process. Previous approaches were limited to defining the interaction of one or a few genes, but the availability of mutants affecting all of the nonessential yeast genes allowed the identification of over 800 interactions in this study. In addition, the synthetic genetic array technique used in this study allowed identification of every nonessential gene in yeast that interacts with an essential replication protein, Dna2p. The comprehensiveness of the approach identified most, if not all, of the pathways in which the multitasking Dna2p participates, in a single experiment. The genomic scale of the study significantly accelerates understanding of this protein over traditional, low-throughput genetic methods.

## Introduction

In order to preserve the fidelity of genome duplication during DNA replication, cells with complex genomes have evolved a network of pathways composed of the DNA replication apparatus, DNA repair proteins, and regulatory activities. Despite years of general characterization, knowledge of the specific mechanisms by which these pathways are integrated to protect the genome is still incomplete because of the complexity of underlying replication fork processes and their regulation. The challenge in understanding high fidelity genome transmission has progressed from identification and characterization of the individual DNA replication components to investigation of how they combine to form pathways orchestrating repair and regulation.

The first line of defense against genome instability resides with the enzymes of the DNA replication apparatus itself. While the most familiar example is the proofreading activity found in the DNA polymerases, other proteins of the replisome have also evolved substrate specificities to address errors made during replication fork progression. One of these proteins is Dna2p, a helicase/nuclease. The *dna2–1* mutation was identified in a screen for yeast mutants defective in DNA replication based on an assay using permeabilized cells [1]. *dna2–1* strains were then shown to accumulate subgenomic-size DNA fragments when incubated at the restrictive temperature [2]. Dna2p has both DNA helicase and single-stranded nuclease activities [3,4]. Biochemical and genetic characterization has revealed that Dna2p is involved in the processing of some, but not all, Okazaki fragments. Specifically, it has been proposed that Dna2p acts with FEN1 to remove RNA primers from Okazaki fragments whose 5′ RNA/DNA termini have been extensively displaced by DNA polymerase (pol) δ. Four lines of evidence support a role for Dna2p in Okazaki fragment processing (OFP). First, Dna2p co-purifies with FEN1, which is a structure-specific nuclease required for OFP in the SV40 in vitro replication system [5,6]. Second, overexpression of the *Saccharomyces cerevisiae* FEN1 gene, *RAD27,* suppresses the temperature-sensitive (ts) growth defect of a *dna2–1* strain, and furthermore, the *dna2–1 rad27*Δ double mutant is synthetically lethal [7,8]. Third, biochemical reconstitution experiments have shown that excessive strand displacement by pol δ creates long 5′ flaps that are cleaved inefficiently by FEN1, and that initial cleavage of these flaps by Dna2p potentiates more efficient subsequent cleavage by FEN1 [9–13]. Finally, Dna2p prefers to act on flaps with secondary structures in vitro, i.e., hairpins or fold-backs containing CTG repeats, which is probably where helicase functionality becomes necessary [12]. Thus, the Dna2p nuclease has evolved the ideal mechanism for highly specific action at a replication fork, requiring the presence of an unpaired 5′ terminus for activity, and displaying a complete lack of activity for single-stranded gaps in duplex DNA, which would result in recombinogenic double-strand breaks (DSBs). Nevertheless, there is a real question of whether the excessively displaced flaps used to study Dna2p in vitro ever occur in vivo, and some biochemical evidence suggests this may not be the case. Therefore, although biochemical data indicate that Dna2p is required for OFP when FEN1 or pol δ activity is impaired, identification of Dna2p's in vivo substrates requires more appropriate genetic and physiological assays than have been applied to date [14].

The second and third lines of defense for preventing genome instability during DNA replication are the DNA repair pathways and regulatory pathways, such as cell cycle checkpoints. Increasing evidence suggests that these pathways are integrated into the replication pathway through their use of certain replication proteins [15]. Dna2p seems to be one of these multitasking proteins. Besides its function in OFP, our evidence strongly suggests that Dna2p provides a link between DNA replication and DNA repair, since *dna2* mutants are sensitive to methyl methane sulfonate (MMS), X-rays, bleomycin, and hydroxyurea (HU) [8,16,17].

To provide a comprehensive view of the roles of Dna2p at the replication fork and in other genome maintenance pathways, we conducted a large-scale synthetic lethal screen by synthetic genetic array (SGA) analysis using *DNA2* as a query [18,19]. Two genes are synthetically lethal if single mutants, defective in either gene, are viable, while double mutants, defective in both genes, are inviable. Two mutants are synthetically sick if the double mutant grows significantly slower than either single mutant. Synthetic lethality is useful for identifying redundancy and complementarity, e.g., pathways that compensate for functional deficiencies in each other or genes encoding products that are both required to efficiently process a common substrate, which is often the case for DNA replication and repair proteins. Synthetic lethal screens not only reveal previously unknown genetic interactions with queried mutants but also how gene products and their corresponding pathways functionally associate.

Our results provide a catalogue of most, if not all, pathways that are interdependent with or require Dna2p, thus revealing both the extent and limits of its multitasking character. The work not only confirms a role in OFP, but also identifies functions in (1) a replication/repair helicase subnet, (2) DSB repair and mismatch repair, (3) the replication stress checkpoint, (4) sister chromatid cohesion, (5) chromatin dynamics, (6) histone modification, and (7) osmotic and oxidative stress responses. In a more general sense, the interactions link a specific network of DNA repair and regulatory pathways to a specific network of replication genes that together maintain high fidelity lagging-strand DNA replication.

## Results

### SGA Screens

Since *DNA2* is an essential gene, either a conditional or hypomorphic allele is required for a synthetic lethal screen. We chose two alleles, *dna2–1,* a ts mutant sensitive to a variety of DNA damaging agents, and *dna2–2,* a mutant that grows at 23 °C and 37 °C, but is sensitive to MMS, bleomycin, and X-rays [8,20]. *dna2–1* contains a P504S substitution in a region of the protein N-terminal to both the nuclease and helicase domains [3]. All enzymatic activities of the Dna2–1 protein are reduced relative to wild-type (WT)—DNA-stimulated ATPase, DNA helicase, and single-stranded DNA nuclease activity [21]. The *dna2–2* mutation changes arginine at position 1235, an invariant residue in helicase region IV, to glutamine [8]. A crystal structure of *PCR* helicase shows that residues in region IV bind the adenine base of ATP [22]. The *dna2–1* strain grows very slowly, even at the permissive temperature, so synthetic sickness is sometimes difficult to unambiguously assign for this mutant. The *dna2–2* mutant grows faster than the *dna2–1* mutant, and synthetic sickness, characterized by slow growth, can be assigned with greater confidence using *dna2–2* strains. The use of each *dna2* allele in a separate SGA screen expands the range of detectable synthetic lethal interactions.

Table 1 lists the validated synthetic lethal and synthetic sick interactions of *dna2–1* and *dna2–2* strains obtained from the SGA screen (see Materials and Methods). Validation was performed by preparing a new heterozygous diploid between the respective *dna2* allele and the candidate gene, followed by sporulation and tetrad dissection. The *dna2*-allele specificity of some of the interactions may turn out to be significant, as the mutations differentially affect helicase and nuclease activity. Mutants that appeared to show growth defects in the primary screen but that did not meet the stringent requirements (see Materials and Methods) for interaction imposed by the secondary tetrad analysis are found in Table S1. Mutants synthetically lethal or synthetically sick with either *dna2–1* or *dna2–2* fall into the following categories: genes involved in OFP *(rad27*Δ, *exo1*Δ, *yen1*Δ, *rnh35*Δ, *rnh202*Δ, *pol3–01, rpa1, elg1*Δ, *pol1,* and *pri1),* nonessential helicases involved in maintaining chromosome stability (*sgs1*Δ, *srs2*Δ, and *rrm3*Δ), genes involved in repair *(rad52*Δ, *mre11*Δ, *rad50*Δ, *xrs2*Δ, *sae2*Δ, *mms1*Δ, *mms22*Δ, *slx5,* and *slx8),* genes involved in the DNA replication checkpoint (*mrc1*Δ, *csm3,* and *tof1*Δ), genes involved in chromosomal cohesion (*ctf4*Δ and *ctf18*Δ), genes involved in chromatin disassembly/assembly and nucleosome modification and remodeling (*spt16, pob3*, *rad6*Δ, *bre1*Δ, *swd1*Δ, *swd3*Δ, *hst3*Δ, *rpd3*Δ, *pho23*Δ, *rtf1*Δ, and the histone chaperone *asf1*Δ), genes involved in the oxidative stress response (*lys7*Δ and *sod1*Δ), a gene in the osmotic stress response (*hog1*Δ), and genes involved in degradation of short-lived proteins (*ubc4*Δ), in polarized cell growth (*cla4*Δ), and in mRNA processing (*trf4*Δ and *rtt103*Δ). Previous studies showed *dna2* to be synthetically lethal with several essential genes: *mcm10–1,* involved in initiation of replication [23]; *cdc9,* a DNA ligase ([24], although see also [8]); *rpa1,* a single-stranded DNA binding protein [13]; and *spt16* and *pob3,* two genes involved in chromosome remodeling [8].

**Table 1:**
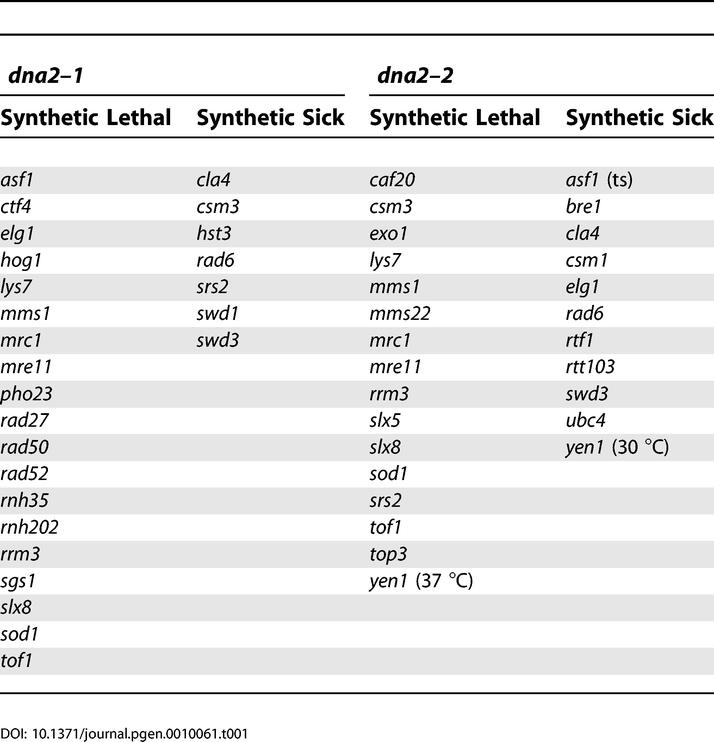
Genes Identified as Putative *DNA2* Interacters from the SGA Screen with *dna2–1* and *dna2–2* Queries and Verified by Tetrad Dissection

**Figure 1:**
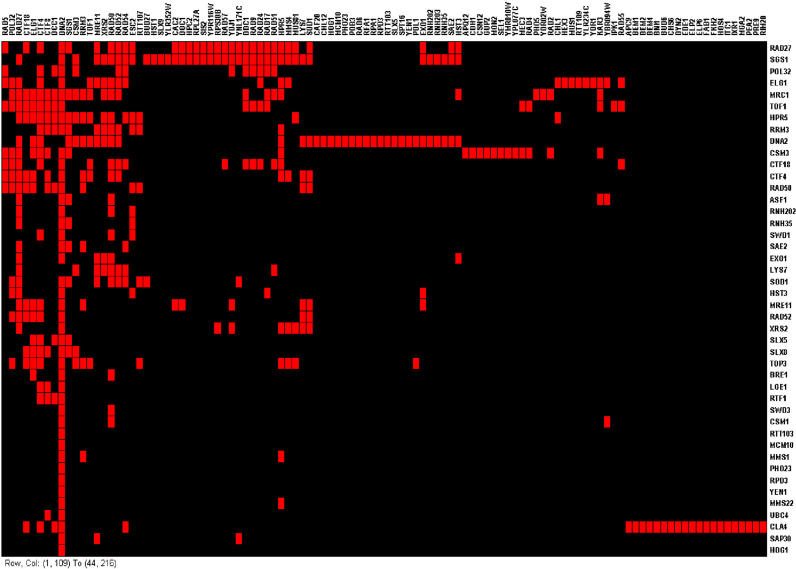
Clustering Analysis Hierarchical two-dimensional clustering analysis was applied to the *DNA2* interactions and those of 43 genes synthetically lethal with *DNA2* (see Table 1). The interactions were clustered with respect to the results of the current screen and 43 previous screens using these genes as queries. A total of 322 genes and 876 interactions, each indicated by a red box, were identified. This panel shows a zoom into the region of most significant overlap of shared genetic interactions. In addition to including reduced fitness genes, this analysis includes genes that give increased fitness with *dna2,* such as *pol32*Δ.

**Figure 2:**
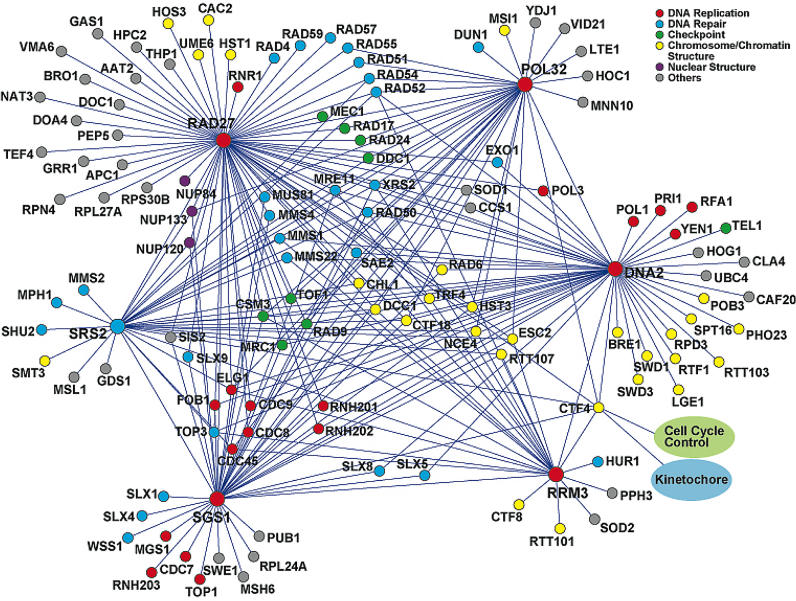
A Genome Stability Network Data were compiled with the Payek program using the *dna2* screen results in Table 1 and the results of the previous screens [18,19,92], as well as data compiled for candidate genes synthetically lethal with *rrm3*Δ [47,75]. The data are presented in tabular form in Table S2.

The total number of genes that interact with *dna2* to date is 56, a connectivity similar to that found in previous screens [18]. Figure 1 shows two-dimensional hierarchical clustering of the data from the current screen and that of previous screens using 43 genes that interact with *DNA2* as queries [18]. Figure 2 provides a graphical representation of the network (consisting of 322 genes and 876 interactions) in which *DNA2, RAD27, SGS1, SRS2 (HPR5), RRM3,* and *POL32* form the six major hubs (largest number of connections). We were surprised that none of these genes showed interactions with cell cycle genes. *ctf4*Δ, however, which is synthetically lethal with all six of these hub genes and many of the other nodes as well (Figure 2), is synthetically lethal with *clb2*Δ, *clb3*Δ, *clb5*Δ, *mad1*Δ, *mad2*Δ, *mad3*Δ, *bub1*Δ, *bub2*Δ, and *bub3*Δ [18]. Thus, *CTF4* may provide a missing link between hub genes and cell cycle and kinetochore functions. The same information is shown in tabular form in Table S2, which contains the interacting genes organized by functional categories defined in the MIPS database (http://mips.gsf.de/genre/proj/yeast/index.jsp). Mutations (identified in studies reported below) that suppress *dna2* alleles are also included. We propose that the genes showing the greatest number of interactions encode products that share at least one substrate or have at least one overlapping function.

### Okazaki Fragment Processing

Although Dna2p is commonly considered to be required for OFP*,* this role is far from established. We were therefore interested to find that the SGA experiments identified interactions between *DNA2* and additional structure-specific nucleases that, like FEN1, are thought to be involved in overlapping OFP pathways. First, *dna2–1* was found to be synthetically lethal with mutations in genes encoding two subunits, *rnh35*Δ and *rnh202*Δ, of the main RNaseH in yeast [25], suggesting that Dna2p may be involved in an OFP pathway redundant with RNaseH2.

**Figure 3:**
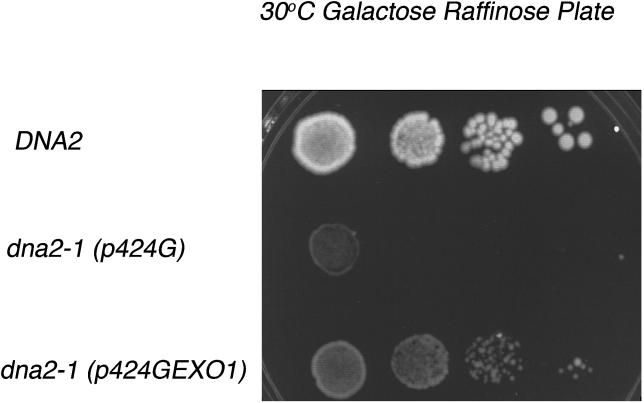
Suppression of *dna2* by ExoIp Overexpression Strain W303 *dna2–1* was transformed with the plasmids pRS424G and pRS424G-EXO1, an empty plasmid vector and a plasmid expressing ExoIp from the *GAL110* promoter, respectively. W303RAD5 strains *dna2–1 7A(pRS424G)* and *dna2–1(pRS424G-EXO1)* were grown to mid log phase and serially diluted on yeast-dextrose galactose- and raffinose-containing plates and incubated at 30 °C for 5 d.

Second, *dna2–2* was synthetically lethal with mutations in genes encoding two exo-endonucleases structurally related to *RAD27, exo1*Δ and *yen1*Δ. *EXO1* is thought to provide backup function for FEN1 in OFP, since *rad27*Δ *exo1*Δ is synthetically lethal (in some genetic backgrounds) and since overexpression of *EXO1* suppresses the ts growth of *rad27*Δ mutants [26–28]. Overexpression of *EXO1* also suppressed the temperature sensitivity of *dna2–1* at the restrictive temperature of 30 °C (Figure 3), though not at 37 °C [21]. Exo1p nuclease acts on 5′ flap-containing structures, and these genetic interactions suggest that such flaps may be the common in vivo substrate of both Dna2p and Exo1p [29].

We also found that *dna2–2* is synthetically sick with *yen1*Δ at 30 °C and lethal, i.e., ts, at 37 °C. Yen1p shows 23% identity with Rad27p, 33% identity with Rad2p (founding member of this nuclease family and involved in nucleotide excision repair), and 24% identity with Din7p (Rad2p-like endonuclease proposed to be involved in mitochondrial mismatch repair). *yen1*Δ mutants do not appear to have DNA damage or growth defects, and a *yen1*Δ *exo1*Δ *din7*Δ *rad2*Δ mutant has no growth defect at either 30 °C or 37 °C [26]. Synthetic lethality with *dna2* is the first informative phenotype reported for *yen1*Δ, although *yen1* is synthetically lethal with another gene that is in turn synthetically lethal with several replication mutants (see Protocol S1).

Elg1p, like Dna2p, is proposed to be involved in OFP, as well as in telomere silencing and length regulation [17,30,31]. *elg1*Δ *dna2–1* mutants are synthetically lethal (Table 1). Elg1p is homologous to Rfc1p (the large subunit of the replication factor C clamp loader), to Rad24p (a Rfc1p homolog required for DNA damage checkpoints), and to Ctf18p (another Rfc1p homolog involved in chromosomal cohesion). *dna2–2* is also synthetically lethal with *ctf18*Δ [8], but not with *rad24*Δ (this work). S phase progression in *elg1*Δ mutants is slower than in WT strains, suggesting that Elg1p is involved in replication fork translocation [30–32].

### Interaction of *dna2* with Pol δ

**Figure 4:**
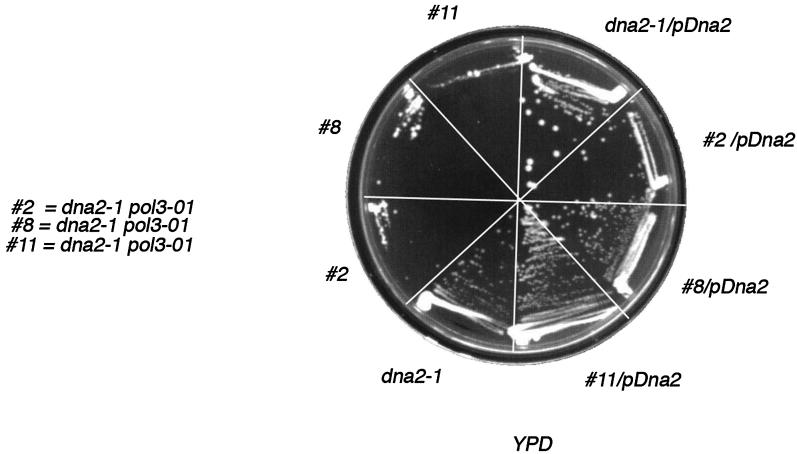
Synthetic Lethality Between *dna2* and *pol3–01* Strain W303 *dna2–1* carrying a *TRP1 CEN3 DNA2* plasmid was transformed with an integrating *URA3 pol3–01* plasmid [120] cut with BamH1. The transformants were streaked on 5-FOA medium to excise the WT *POL3* gene and identify clones with the *pol3–01* mutation. Three transformants carrying the *pol3–01* mutation (isolates 2, 8, and 11) were restreaked on YPD medium containing 2-amino-5-fluorobenzoic acid (FAA), which selects for strains that have lost the *DNA2 TRP1*–containing plasmid [121]. Three *dna2–1 pol3–01* colonies showing some residual growth on the FAA plates were restreaked on YPD-containing medium. The same three isolates of *dna2–1 pol3–01* but containing the *DNA2 TRP* plasmid, i.e., that had not been grown in the presence of FAA, are shown as controls, as indicated.

It has been proposed, based on in vitro biochemical reconstitution in vitro, that not all Okazaki fragments require Dna2p for processing. Instead, the in vivo substrates of Dna2p are only those Okazaki fragments on which pol δ produces 5′ flaps longer than 30 nucleotides [10,12,14,33]. We wished to devise a genetic test of this hypothesis. One way to do so is by generating excessive strand displacement in vivo and assessing whether there is then an increased requirement for *DNA2*. This was accomplished by testing the viability of a strain containing both the *dna2–1* mutation and *pol3–01,* a mutation in pol δ known to increase strand displacement in vitro [33]. As shown in Figure 4, *dna2–1 pol3–01* is synthetically lethal. We propose that excessive strand displacement in vivo in the *pol3–01 dna2–1* strain causes the lethality, supporting the predictions from purely biochemical reconstitution.

**Figure 5:**
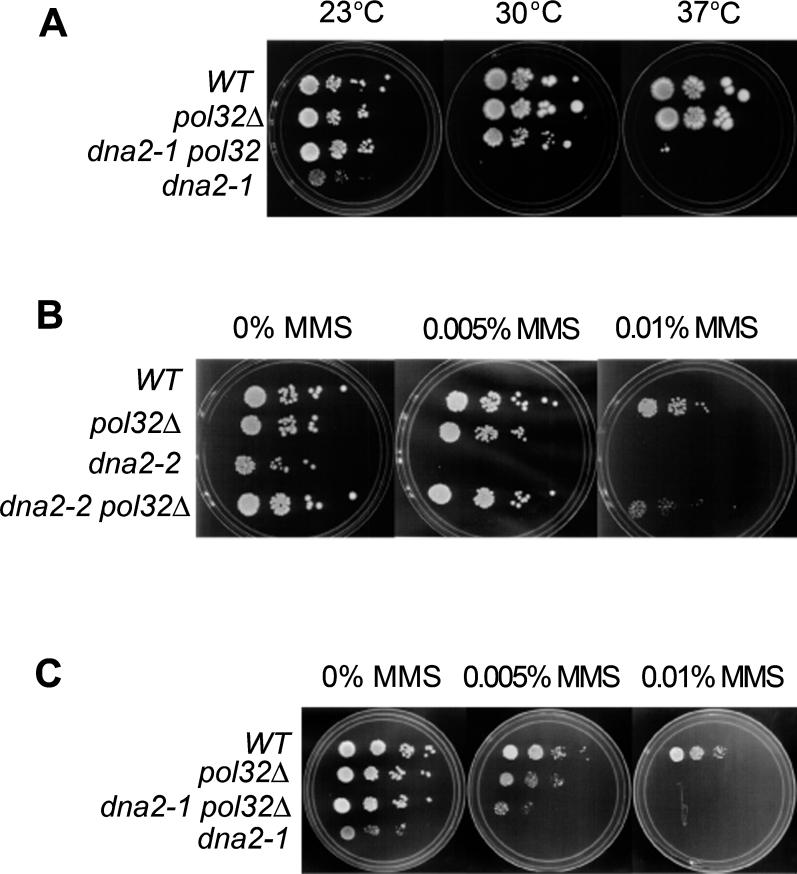
Suppression of Slow Growth and MMS Sensitivity of *dna2* Mutants by *pol32*Δ (A) WT*, pol32*Δ, *dna2–1 pol32*Δ, and *dna2–1* strains were grown to log phase, serially diluted, and plated on YPD plates and incubated at 23 °C, 30 °C, and 37 °C for 5 d. (B) WT, *pol32*Δ, *dna2–2,* and *pol32*Δ *dna2–2* strains were grown to log phase, serially diluted, and incubated on MMS-containing YPD plates for 3 d at 30 °C. (C) WT*, pol32*Δ, *dna2–1 pol32*Δ, and *dna2–1* strains were grown to log phase, serially diluted, and grown on MMS-containing YPD plates for 5 d at 23 °C. All strains are isogenic with strain 4741 (Table S4). (*dna2–1* grows slowly even at 23 °C, and plates at 23 °C were photographed before they were fully grown so that the other strains would not be overgrown.)

Another pol δ subunit mutant, *pol32*Δ, is synthetically lethal with *rad27*Δ, and the network of synthetic lethal interactions of *pol32*Δ is similar to that of *rad27*Δ, *rrm3*Δ, *sgs1*Δ, and *srs2*Δ, which are also synthetically lethal with *dna2* (See Figure 2). We were surprised that *pol32*Δ did not show up in our SGA screen, and directly tested *dna2–1 pol32*Δ for synthetic lethality. Rather than showing synthetic lethality, *pol32*Δ suppressed the slow growth phenotype of the *dna2–1* strain at 23 °C and suppressed the ts growth phenotype of the *dna2–1* strain at 30 °C (Figure 5A). The *pol32*Δ mutation did not suppress the lethality of the *dna2–1* strain at 37 °C (Figure 5A) or the lethality of a *DNA2* deletion (not shown). The *pol32*Δ deletion mutation also suppressed the DNA damage sensitivity of the *dna2–1* and *dna2–2* mutants (Figure 5B and 5C, respectively). *pol32*Δ mutants are sensitive to different concentrations of MMS than are *dna2* mutants. *dna2–1* and *dna2–2* mutants are sensitive to 0.005% MMS, while *pol32*Δ strains are resistant to 0.005% MMS but are sensitive to 0.01% MMS. Both *dna2–1 pol32*Δ and *dna2–2 pol32*Δ mutants showed MMS sensitivity similar to *pol32*Δ strains rather than to *dna2–1* or *dna2–2* strains (Figure 5B and 5C). Pol32p is a nonessential subunit of the pol δ holoenzyme and is required for full processivity of pol δ [34]. Therefore, a less processive pol δ suppresses the growth and repair defects of both nuclease- and helicase-deficient mutants of *dna2,* suggesting that Dna2p is acting on flaps arising from excessive strand displacement by pol δ during DNA repair as well as during OFP. The synthetic lethality of *dna2* and *pol3–01* and the suppression of *dna2–1* by *pol32*Δ provide strong genetic evidence that the Dna2p substrates shown to be optimal in vitro are also substrates in vivo (see Discussion).

### The Helicase Network

We have reported previously that *dna2–2* is synthetically lethal with *sgs1*Δ, *srs2*Δ, and *rrm3*Δ [35–37], and our SGA screen also detected these genes. More significantly, there is extensive overlap between genes that are synthetically lethal with *dna2* and those that are synthetically lethal with *sgs1*Δ and *srs2*Δ, as shown in Figures 1 and 2.

To assess the functional relationship between Dna2p and these helicases, we have tried to further determine whether the synthetic lethality between *dna2* and the helicase genes shown in Table 2 stems from defects in DNA replication per se or from the aberrant DNA structures that arise during repair of DNA replication errors. Towards this end, we first checked whether the lethality was suppressed by mutations in genes considered necessary for resolving potentially lethal intermediates that form when the original lesions enter the recombination pathway for repair [38]. The results of our current analysis of *dna2–2 sgs1*Δ, *dna2–2 srs2*Δ, and *dna2–2 rrm3*Δ are summarized in Table 2. *dna2–2 sgs1*Δ and *dna2–2 srs2*Δ were synthetically lethal and were either inefficiently or efficiently suppressed, respectively, by *rad51*Δ, as we reported previously ([35,37]; see Discussion). New here is the finding that *dna2–2 rrm3Δ* lethality was not suppressed by mutations in the recombination pathway (Table 2). This indicates that cell death in *dna2–2 rrm3*Δ does not result from the accumulation of nonresolvable recombination intermediates but from the formation of early replication intermediates, or possibly blocked DNA replication forks.

**Table 2:**
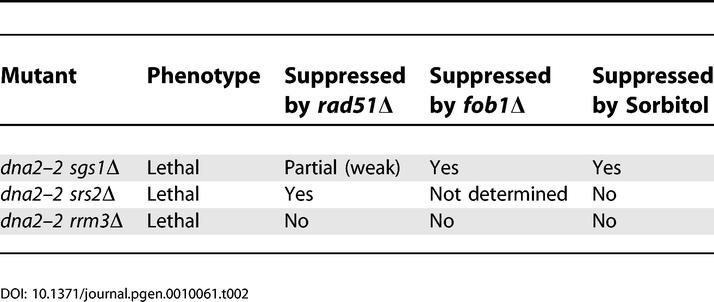
Genetic Interactions of *DNA2* with Other Helicases

Gene pairs encoding three different heterodimeric complexes *(MMS4/SLX3, SLX1/4,* and *SLD5/8),* although they are not DNA helicases, are required for viability in the absence of *SGS1* and, thus, are part of the helicase network [39]. We find that only one of these complexes, defined by *slx5*Δ and *slx8*Δ, shows synthetic lethal interactions with *dna2*. Since the function of the Slx5/8p complex is unknown, we cannot yet predict the nature of the substrates that might be shared with Dna2p.

### DNA Repair

We have previously shown that *dna2* and *rad52*Δ mutants are synthetically sick [16]. *rad52*Δ also appeared synthetically sick with the *dna2* alleles in the SGA screen. We were surprised that other genes required for recombinational repair did not appear synthetically lethal with *dna2* in the SGA screen, since they are synthetically lethal with *rad27*Δ. To insure that we had not missed such genes, *dna2–1 rad51*Δ and *dna2–1 rad55*Δ, as well as *dna2–2 rad51*Δ and *dna2*-2 *rad59*Δ, mutants were constructed. Consistent with the global screen, these double mutants did not appear to be synthetically lethal or sick. We also found that *dna2–2 rad51*Δ *rad59*Δ triple mutants were viable (not shown). Our results indicate that the Rad51p/Rad59p portion of the Rad52p pathway does not buffer Dna2p function. *MMS1* and *MMS22* are two additional presumed DNA repair genes that are also required for normal S phase progression and that show synthetic lethality with *dna2* mutants in the SGA screen, as previously described [40]. The Mms1p/Mms22p system may correspond to the pathway that results in *dna2–2 rad52*Δ growth defects.

Previously, we reported that *dna2–2* and *rad50–5* double mutants were viable and epistatic for repair [16]. Therefore, one of our most unexpected findings was that *dna2 mre11*Δ and *dna2 rad50*Δ strains are synthetically lethal (see Table 1). *rad50–5* is a point mutation of *RAD50* that is as sensitive to irradiation as either a *rad50*Δ or a *rad52*Δ strain. Clearly, some function must remain in the point mutant as compared to the deletion mutant, however, since the *dna2–2 rad50–5* strain is viable while the *dna2–2 rad50*Δ strain is inviable. *MRE11, RAD50,* and *XRS2* encode members of the Mre11p complex, which is required for the intra-S phase checkpoint, for homologous recombination, for non-homologous end joining, and for telomere maintenance ([41,42] and references therein). Although *xrs2*Δ was not found in our SGA screen, we have since shown that *dna2–2 xrs2*Δ is synthetically lethal (this work).

### *dna2* Mutants Do Not Require the *rad9, mrc1, mec1,* or *tel1* Checkpoint Activities for Viability

Our analysis of the interaction between *dna2* and checkpoint mutants supports the view that *dna2–1* and *dna2–2* mutants accumulate less damage than *rad27*Δ mutants. First, we found that neither *dna2* allele used in our screen is synthetically lethal with *rad9,* a mediator of the intra-S DNA damage checkpoint, as found previously [8,43] for other *dna2* alleles. *rad27*Δ mutants are synthetically lethal with *rad9*Δ*.* In addition, *rad27*Δ mutants are synthetically lethal with the DNA damage checkpoint mutants *rad24*Δ and *rad17*Δ, which represent the checkpoint clamp-loader-like complex and the checkpoint clamp-like proteins, respectively, but *dna2 rad24*Δ and *dna2 rad17*Δ are both viable (this study). Thus*, dna2* mutants do not accumulate sufficient damage to require the intra-S DNA damage checkpoint for viability at the permissive temperature.

**Figure 6:**
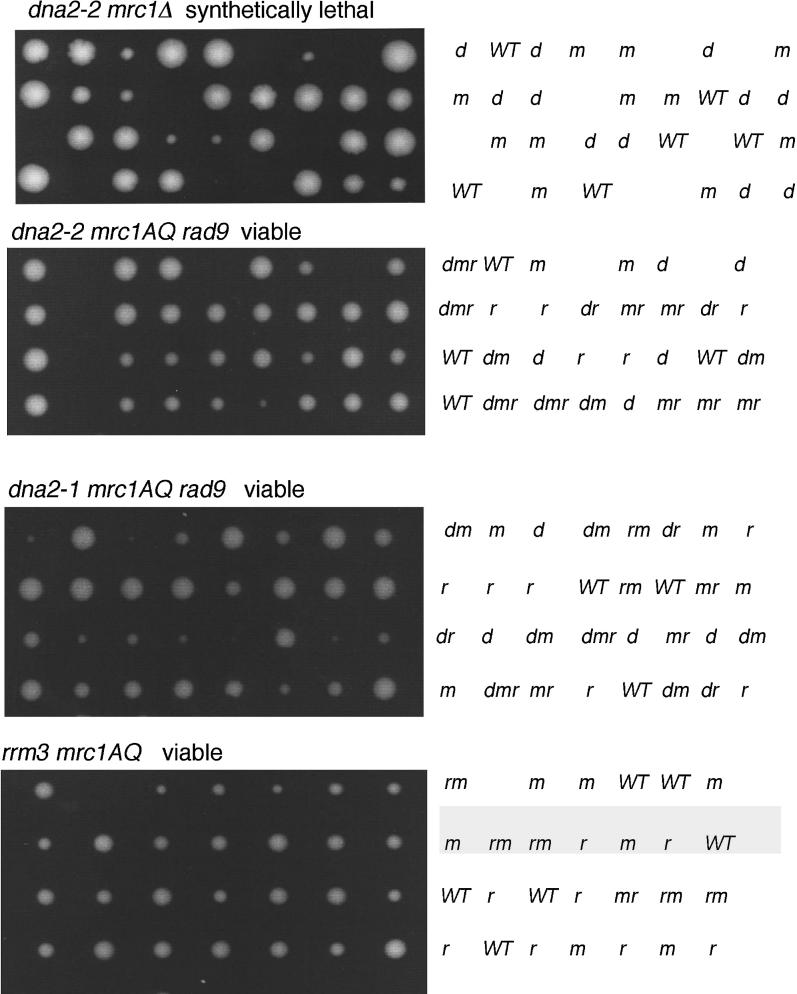
Separation-of-Function Checkpoint Mutants *mrc1AQ* and *rad9*Δ Are Not Synthetically Lethal with *dna2* or *rrm3*Δ Mutants *mrc1*Δ experiments were carried out in the isogenic 4741 strain and are listed in Table S4. *mrc1AQ* experiments were carried out in strains isogenic with W303 *RAD5* ^+^. Panel 1 (top): segregants from a *DNA2/dna2–2 MRC1/mrc1*Δ diploid. Segregants are isogenic with *4741*. *d, dna2–2; m, mrc1*Δ*.* Panel 2: segregants from a *MRC1/mrc1AQ RAD9/rad9*Δ *DNA2/dna2–2* diploid. *d,* *dna2–2; m, mrc1AQ; r, rad9*Δ. Panel 3: segregants from a *DNA2/dna2–1 MRC1/mrc1AQ RAD9/rad9*Δ strain. *d, dna2–1; m, mrc1AQ; r, rad9*Δ. Panel 4 (bottom): segregants from a *RRM3/rrm3*Δ *MRC1/mrc1AQ* diploid. *m, mrc1AQ; r, rrm3*.

We found in the SGA experiments, however, that *dna2* mutants are synthetically lethal with *mrc1*Δ, *tof1*Δ, or *csm3*Δ (Table 1). These genes define a checkpoint pathway thought to operate in parallel with the Rad9p pathway to respond specifically to replication stress. Mrc1p, Tof1p, and Csm3p are required for Rad53p phosphorylation in the presence of HU or MMS, both of which induce S phase DNA damage [18]. Mrc1p is an adapter molecule in the checkpoint, whose phosphorylation by Mec1p at SQ/TQ motifs is required for activation of downstream effectors [44,45]. *dna2 mrc1*Δ synthetic lethality seemed to contradict the observation that other *dna2* alleles actually show improved survival in the absence of the checkpoint ([43]; see below), and suggested that such synthetic lethality might be due to another function of *MRC1*. *MRC1* and *TOF1* appear to play a direct role in yeast DNA replication, as well as in the checkpoint. *mrc1*Δ strains also show a slow S phase, and Mrc1p and Tof1p have been localized to moving replication forks [46]. Osborn and Elledge [45] constructed a separation-of-function *mrc1* mutant that has all 17 TQ and SQ Mec1p target sites mutated to non-phosphorylatable AQ. This mutant, *mrc1AQ*, like *mrc1*Δ, is checkpoint defective, as evidenced by the fact that Rad53p phosphorylation is blocked in *mrc1AQ rad9*Δ mutants upon treatment with HU or MMS. However, *mrc1AQ* mutants are replication proficient. The *mrc1AQ* mutant allowed us to ask whether the replication defect of *mrc1*Δ was responsible for *dna2 mrc1*Δ synthetic lethality. *dna2–1 mrc1AQ* and *dna2–2 mrc1AQ* strains were constructed and were viable and did not appear synthetically sick, indicating that the checkpoint function of Mrc1p is not required for viability in *dna2* mutant backgrounds (Figure 6). In order to be sure that *RAD9* was not substituting for *MRC1* in the *dna2–2 mrc1AQ* mutant, *dna2–2 mrc1AQ rad9*Δ mutants, which lack both the DNA damage and replication stress checkpoints, were constructed (Figure 6). The viability of *dna2–2 mrc1AQ rad9*Δ strains suggests that it is inactivation of the replication function of *MRC1* that is responsible for synthetic lethality in the *dna2 mrc1*Δ mutants.

*mrc1*Δ and *rrm3*Δ mutants are also synthetically lethal [47] and, because *RRM3* and *DNA2* interact (see Table 2), we asked whether the *mrc1*Δ *rrm3Δ* synthetic lethality is caused by a checkpoint or replication defect. The *mrc1AQ rrm3*Δ double mutants showed the same viability as the single mutants (Figure 6). Since *dna2* and *rrm3* are also synthetically lethal, this suggests that *DNA2*, *RRM3,* and *MRC1* functions may be interdependent in DNA replication.

**Figure 7:**
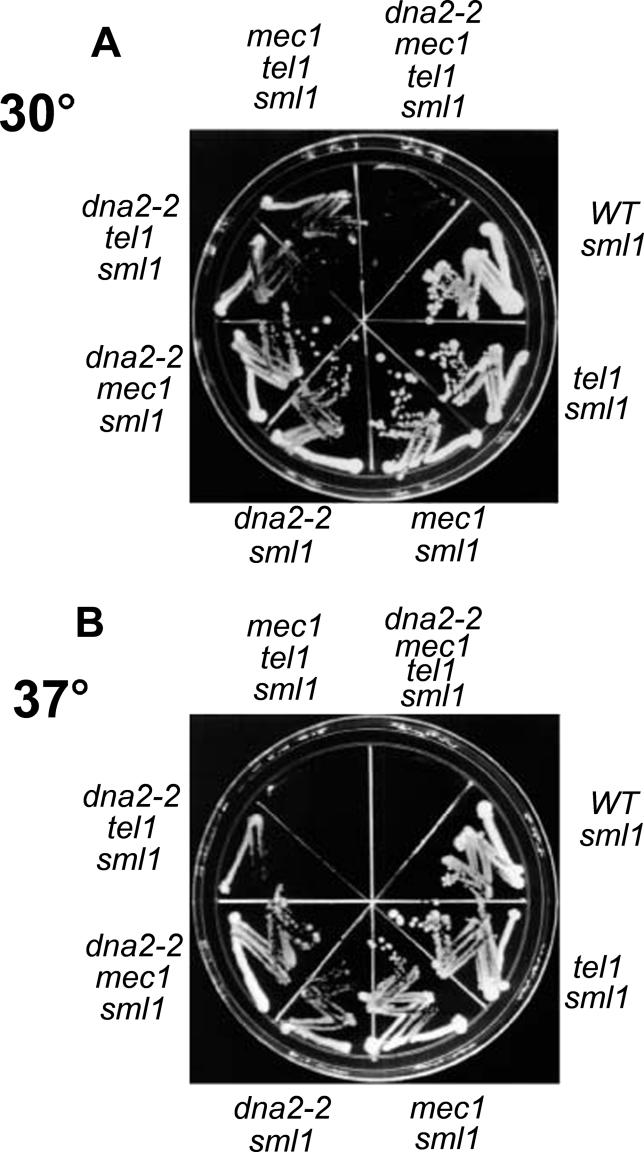
Synthetic Lethality of *mec1*Δ *tel1*Δ with *dna2–2* Mutations Strains used in these experiments are listed in Table S4, and were isogenic or congenic with W303 *RAD5* ^+^. Segregants of a *MEC1/mec1*Δ *TEL1/tel1*Δ *DNA2/dna2–2 SML1/sml1Δ* diploid were placed on a YPD plate incubated at 30 °C (A) or 37 °C (B).

To further test the idea that S phase checkpoint signaling is dispensable for *dna2* mutant viability, the interaction of *dna2* with *mec1Δ* and *tel1Δ*, mutations in genes upstream of *MRC1* and *RAD9* in the checkpoint, were investigated. A *dna2–2/DNA2 tel1*Δ/*TEL1 mec1*Δ/*MEC1 sml1*Δ/*SML1* heterozygote was sporulated, and Table S3 lists the genotypes obtained among the tetrads. (*sml1*Δ allows for *mec1*Δ viability.) The *mec1*Δ mutation partially suppressed the slow growth phenotype of *dna2–2* strains. Thus, as previously observed for *dna2–20* [43], the Mec1p-mediated checkpoint is deleterious in the *dna2–2* mutant (Figure 7). The *tel1*Δ mutation shows negative synergy but not lethality with *dna2–2* at 37 °C (Figure 7). The negative synergy between *dna2–2* and *tel1*Δ is evidence that Dna2p and Tel1p may function together at DSBs and/or at telomeres, along with the Mre11p complex [48]. *dna2–2 tel1*Δ *mec1*Δ triple mutants were recovered. As shown in Figure 7, however, the *dna2–2 tel1*Δ *mec1*Δ mutant grew more slowly than any of the single or double mutants. The telomere defects of the *mec1*Δ *tel1*Δ strain caused it to senesce as rapidly as *est2*Δ (telomerase catalytic subunit deleted) strains [49]. The *dna2–2* mutation may cause additional defects in telomere replication. Thus, enhanced telomeric senescence might account for the slow growth of the *dna2–2 tel1*Δ *mec1*Δ mutant, just as we have shown that *dna2–2 est2*Δ is synthetically lethal due to accelerated senescence [17].

Finally, we tested the interaction of *DNA2* with the checkpoint effector kinase *RAD53*. We crossed *dna2–1* and *dna2–2* to an isogenic *rad53*Δ *sml1*Δ strain. *dna2–1 rad53*Δ *sml1*Δ and *dna2–2 rad53*Δ *sml1*Δ mutants were fully viable. The viability of *dna2 rad53* strengthens the conclusion that the DNA damage arising in a *dna2* mutant is not sufficient to require the S phase checkpoint for viability. (*dna2–1* mutants, however, do induce an amount of damage above the threshold for checkpoint activation at restrictive temperatures, since they arrest at the metaphase to anaphase transition in a *MEC1*-dependent manner [M. E. B. and J. L. C., unpublished data].)

### Sister Chromatid Cohesion and Repair of DSBs in the rDNA

**Figure 8:**
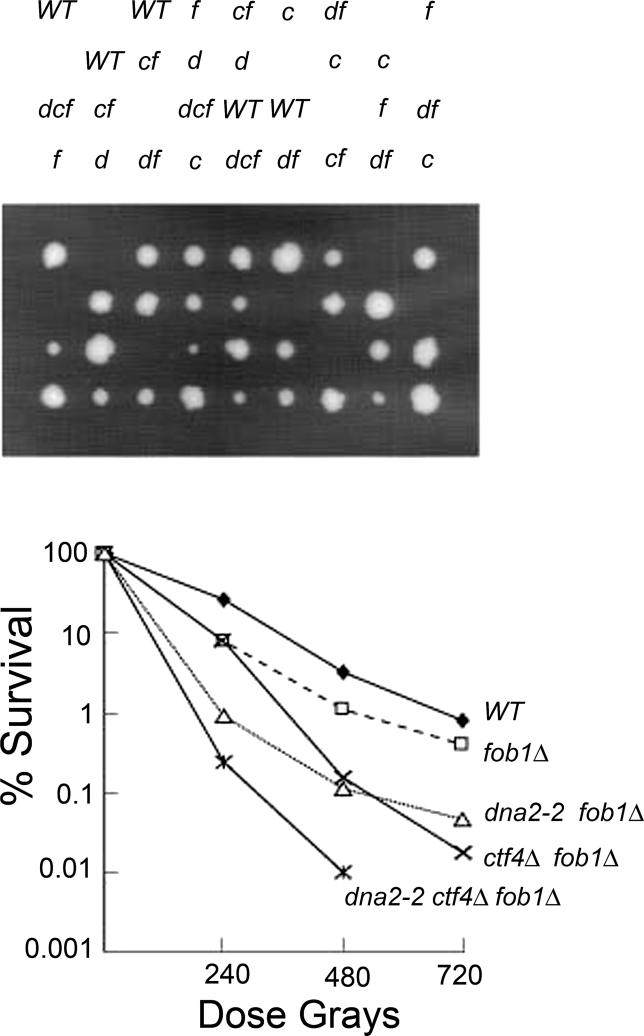
Deletion of *FOB1* Suppresses *dna2 ctf4*Δ Synthetic Lethality Top panel: Tetrads from the dissection of a *DNA2*/*dna2–2 CTF4*/*ctf4*Δ *FOB1/fob1*Δ heterozygote. *c, ctf4*Δ; *d, dna2*Δ; *f, fob1*Δ; WT, *DNA2 CTF4 FOB1*. The following spores were recovered: 11 WT, 21 *dna2–2*, 26 *fob1*Δ, 17 *ctf4*Δ, 18 *dna2–2 fob1*Δ, 20 *ctf4*Δ *fob1*Δ, zero *ctf4*Δ *dna2–2*, and six *dna2–2 ctf4*Δ *fob1*Δ. Since the triple mutant grew slowly at 30 °C, another 27 spores were dissected and incubated at 23 °C. The following spores were recovered: 14 WT, nine *fob1*Δ, 14 *ctf4*Δ, seven *dna2–2,* 14 *dna2–2 fob1*Δ, nine *ctf4*Δ *fob1*Δ, zero *dna2–2 ctf4*Δ, and nine *dna2–2 ctf4*Δ *fob1*Δ. The triple mutant did not grow at 37 °C. Bottom panel: X-ray sensitivity of *dna2–2 ctf4*Δ *fob1*Δ. Strains with genotype WT, *fob1Δ*, *dna2–2 fob1*Δ, *ctf4*Δ *fob1*Δ, and *dna2–2 ctf4*Δ *fob1*Δ were grown to log phase, irradiated as described in Materials and Methods, serially diluted, plated on YPD plates, and incubated at 30 °C. Strains are isogenic or congenic with 4741.

Ctf4p is a pol α–binding protein [50], and *ctf4*Δ strains are defective in sister chromatid cohesion [20]. *dna2–2* was identified as a mutant synthetically lethal with *ctf4*Δ, but we have shown that *dna2* mutants are not defective in cohesion [51]. We previously reported that the *dna2*-*2* mutation gave rise to an increased frequency of DSBs at the replication fork barrier (RFB) in the rDNA and that deleting *FOB1,* which is required for pausing at the RFB, suppressed DSB formation. A reasonable explanation for all of these observations is that the DSB damage sustained by *dna2–2* mutants at the RFB might require Ctf4p-mediated sister chromatid cohesion for repair. If so, then one would expect *fob1*Δ to suppress *dna2–2 ctf4*Δ synthetic lethality. We dissected 55 tetrads from a *dna2–2 ctf4*Δ *fob1*Δ heterozygote and incubated the spores at 30 °C. Viable *dna2–2 ctf4*Δ *fob1*Δ mutants were obtained in the expected numbers, demonstrating suppression. Although the *dna2 ctf4*Δ *fob1*Δ triple mutants grew at 23 °C and at 30 °C, they did not grow at 37 °C and were highly sensitive to X-rays (Figure 8). The behavior of the triple mutant indicates that defects in the rDNA locus are critical for some of the phenotypes of *dna2–2,* but that defects elsewhere throughout the chromosome must still occur, giving rise to ts growth and DNA damage sensitivity.

### Nucleosome Remodeling: Dna2p Interacts with Pol α and Primase

Pob3p and Spt16p/Cdc68p form a heterodimer that is a component of the ATP-independent chromatin remodeling activity yFACT [52]. *dna2–2* is synthetically lethal with a non-ts allele of *POB3*, *pob3–21* [52], and various alleles of *spt16* are synthetically lethal or sick with *dna2–2* [53]. Since yFACT may participate in both DNA replication and transcription, to investigate a potential link with the role of Dna2p in replication and/or repair, we took advantage of the observation that yFACT interacts both genetically and physically with pol α [54,55]. This suggested *dna2* might be synthetically lethal with a mutant containing a pol α protein that fails to interact with Pob3p/Spt16p, *pol1–1* (with glycine at position 493) [56]. We established the synthetic lethality of *dna2–2* and *pol1–1* (18 tetrads, 46 viable spores, no double *dna2–2 pol1–1* mutants). The *dna2–2 pol1–1* lethality is allele specific, since *dna2–1* is not synthetically lethal with *pol1–17*, a catalytic site mutant [57] (see Discussion). Although yFACT may also affect transcription and the synthetic effects between *dna2* and yFACT components could be due to reduced transcription of *dna2–2* or of other replication genes in the double mutants, the synthetic lethality of *dna2–2 pol1–1* argues that Dna2p and yFACT may interact during DNA replication.

We went on to investigate genetic interactions between *dna2* and genes encoding other pol α subunits. We found that *dna2–2* is synthetically lethal with a primase subunit mutant, *pri1-M4* (18 tetrads, 51 viable spores, no double mutants). This result further implicates Dna2p in lagging-strand DNA replication, as the *pri1-M4* mutant is defective in elongation [58].

### Chromatin Remodeling and Histone Modification

In addition to clarifying the relationship between *dna2* and the yFACT complex by identifying the *dna2*/*pol1–1* interaction, the SGA screen suggested a role for Dna2p in additional complexes that control assembly and disassembly of chromatin during DNA replication. We found that *dna2* mutants are synthetically lethal or synthetically sick with a*sf1*Δ, defective in a chaperone protein involved in histone deposition at the replication fork. Since *dna2* mutants are defective in OFP, they might also be defective in the rapid reassembly of chromatin behind the replication fork, leading to an accumulation of defective chromatin and lethal DSBs [59].

The SGA screen also revealed that *dna2* mutants are synthetically lethal or sick with *rad6*Δ, *bre1*Δ, *swd1*Δ, and *swd3*Δ (see Table 1). Rad6p is a ubiquitin-conjugating enzyme (E2) required for post-replication repair, the N-end rule, and chromatin modification [60,61]. Rad6p functions with two alternative ubiquitin ligases (E3)—*RAD18* in post-replication repair and *BRE1* in histone H2B ubiquitylation [62–65]. *dna2 rad18*Δ mutants grew normally (this work). By contrast, *dna2* showed synthetic sick interactions with *bre1*Δ and with mutations in two genes that act downstream of *BRE1, swd1*Δ and *swd3*Δ (Table 1). Bre1p-mediated histone H2B ubiquitylation is necessary for histone H3 lysine 4 methylation by Swd1p and Swd3p. These results may indicate that *dna2 rad6*Δ growth defects do not result from defective post-replication repair, but rather from defects in histone modification.

*dna2* is also synthetically lethal with *rtf1*Δ, which is involved in recruitment of Set1p and H2B ubiquitylation. *RTF1* is a member of the PAF1p complex. Since the PAF1p complex is involved in transcription, a trivial explanation of the *dna2 rtf1*Δ lethality could be that transcription of *dna2* or of another replication gene is reduced and indirectly creates the synthetic lethality. If so, then *dna2* should also be synthetically lethal with a *paf1*Δ. However, we found that no *dna2* allele tested was either synthetically sick or lethal with *paf1*Δ. Recent evidence suggests that Rtf1p, which recruits Set1p, affects genome-wide ubiquitylation of histone H2B by a mechanism that is distinct from its function as a transcriptional elongation factor [66], and that would explain how we can find no effect of *paf1*Δ but lethality with *rtf1*Δ.

*dna2* also shows synthetic interactions with mutations in genes encoding histone deacetylases: *hst3*Δ and *pho23*Δ. Rpd3p and Pho23p are components of a histone deacetylase complex that is thought to be involved in regulation of initiation of DNA replication [67,68], and therefore we tested *dna2–2 rpd3*Δ, and found that it was synthetically lethal. *DNA2* is the only replication gene that has shown a genetic interaction with *rpd3*Δ to date (see also Protocol S1). (We wish to emphasize that for all of the chromatin-modifying pathways detected in the screen, we have shown, in studies in preparation for presentation elsewhere, that the effects are not due to the trivial explanation of reduced transcription of the mutant *dna2* genes).

### Other Interactions

Additional nodes in the network (oxidative stress genes, osmotic stress genes, and genes involved in RNA modification/catabolism [mutants *trf4*Δ and *rtt103*Δ]) that are synthetically lethal with *dna2* are described and discussed in Protocol S1. Genes involved in transcription elongation (*CAF20*, for example) have not yet been further analyzed because they may reduce transcription of Dna2p or another replication protein and thus indirectly cause lethality.

## Discussion

We find that 56 genes interact genetically with *DNA2*. Comparison of our results with those of previous synthetic lethal screens using 43 of the *DNA2*-interacting genes defines a set of pathways, all of which are interdependent with *DNA*2 and that form a network for preserving genome stability (see Figure 2). The six major hubs shown in Figure 2 link DNA replication, DNA repair, chromosome dynamics, checkpoints, chromosome structure/chromatin, osmotic stress, oxidative stress, and RNA metabolism. A major link to the cell cycle and the kinetochore occurs through a single gene, *CTF4*. Analysis of mutants that give rise to gross chromosomal rearrangements, the type of damage considered to be the most likely result of replication apparatus failure, identifies the same pathways [69–75]. The comprehensive nature of the SGA screen, however, allows greater insight into the structure of the network that coordinates these events. It is striking that this topology can be superimposed on the prokaryotic DNA replication interactome recently identified using protein–protein interactions [76]. The bacterial genome maintenance network consists of many of the *E. coli* orthologs (i.e., pol III holoenzyme, SSB, RecQ, RecG, SbcB, and RecJ) of the yeast replicative polymerase, its subunits, and the helicases and nucleases that form hubs and major nodes in our genetic network [76]. The common denominator in the diverse approaches was the use of a replication gene or protein as the bait. The parallels between the organisms point to evolutionary conservation in the coordination of processes that protect the genome. We suggest that the complexity of such processes was required for the evolution of large genomes, where the fidelity of the replication apparatus itself could not guarantee a sufficiently high level of accuracy and stability in genome transmission.

Current methods of scoring interactions do not result in identification, in the initial SGA screen, of every interacting gene (see discussions in [18]). To approach completeness, following identification of a single gene in a pathway, e.g., *MRE11,* in the SGA screen, we pursued “traditional” investigation, on a gene-by-gene basis, of other genes in the putative pathway, such as *XRS2*. Similarly, the identification of the Bre1p ubiquitylation pathway was interpreted only after testing downstream genes in the histone H2B modification pathway. This type of comprehensive genetic analysis is a powerful new tool for rapidly characterizing the full complement of processes requiring replication genes that might be coming under analysis for the first time, as well as rendering a coherent picture of years of genetic analysis of other genes. The genetic screen then enables one to rationally design experiments to determine, in molecular terms, the contribution of the replication protein to these processes. The outcome of such secondary analyses of *DNA2* is discussed and interpreted below.

### Stronger Links between Dna2p and OFP during Lagging-Strand Replication: *RNH32, RNH202, EXO1, YEN1, POL3, POL32, POL1*, and *PRI1*

#### Structure-specific nucleases.

Although it has been known for some time that *dna2* and *rad27*Δ are synthetically lethal, convincing genetic interactions between *dna2* and other lagging-strand activities have not been previously identified. As shown by our demonstration in this work of synthetic lethality of *dna2* and *rtf1*Δ and the viability of *dna2* and *paf1*Δ (see Results), synthetic lethality with one gene in a pathway does not prove interaction with other genes in that pathway. Therefore, the identification of so many additional lagging-strand genes in the current study is a matter of some note. The synthetic lethality of *dna2* with the genes encoding structure-specific nucleases *(RNH32, RNH202, EXO1,* and *YEN1)* provides the first evidence, to our knowledge, that RNA may be a substrate of Dna2p in vivo and strengthens evidence, as predicted from our in vitro studies [14], that Dna2p acts primarily if not exclusively on flaps in vivo. It has been known for some time that *rad27*Δ *rnh35*Δ is synthetically sick, but not lethal [77]. Since FEN1 is generally considered the major OFP nuclease, the more significant synthetic lethality of *dna2 rnh35*Δ and *dna2 rnh202*Δ was somewhat unexpected, and suggests that Dna2p also acts in an OFP pathway redundant with RNaseH2. Since Rnh35p does not act on flap-containing structures, the common substrate for RNaseH2 and Dna2p is probably RNA. Alternative to a role in OFP, the *dna2 rnh35*Δ synthetic lethality might reflect a redundant role for RNaseH2 and Dna2p in mRNA processing, an additional function proposed for RNaseH2 [78]. *rnh35*Δ mutants also have very short telomeres [79]. Since Dna2p is required for de novo telomere synthesis and *dna2 est1* and *dna2–2 est2* are synthetically lethal [17], *dna2–1 rnh35*Δ lethality could be related to events at telomeres.

Suppression of *dna2* by *EXO1* overexpression, combined with *dna2 exo1*Δ synthetic lethality, suggests that Exo1p can provide a backup for Dna2p in OFP, with 5′ flaps constituting the common substrate. *exo1*Δ is also synthetically lethal with *mre11*Δ, and it has been proposed that Exo1p participates in the resection of ends at DSBs in preparation for recombinational repair [42]. Exo1p may also be involved in a late step in the Msh2p-dependent mismatch repair pathway and perhaps in telomere end processing [27], which might also account for the *dna2 exo1*Δ lethality. There is no biochemical evidence as yet to show whether Yen1p is a structure-specific 5′ to 3′ nuclease, but this is likely given its similarity to FEN1 and Exo1p. Given that Yen1p is a preferential substrate of the Clb5p cyclin-dependent kinase required for proteins that function in G1 and early S phase [80], the *yen1*Δ *dna2–2* synthetic interaction may reflect a direct link between *YEN1* and DNA replication (see also Protocol S1). Since there are about 100,000 Okazaki fragments (1.5 × 10^7^ bp per genome/100 bp per Okazaki fragment) during S phase, it is not surprising that multiple nucleases are involved in OFP.

#### Pol δ.

Our genetic analysis supports the notion that Dna2p processes a specific subset of Okazaki fragment flaps—namely, those arising from excessive strand displacement by pol δ. We showed not only that *dna2* is synthetically lethal with a mutant of pol δ that gives abnormally high levels of strand displacement, but also that *dna2* is suppressed by a mutant that gives decreased strand displacement. *pol3–01* is a mutation that inactivates the 3′ to 5′ exonuclease function of pol δ. This nuclease has three known functions [81]. First, because *pol3–01* is a strong mutator, we can surmise that one function is in proofreading during DNA polymerization. Second, negative synergy of *pol3–01* with *msh2*Δ and *exo1*Δ suggests a role in mismatch repair. These two activities are probably not relevant to the *dna2 pol3–01* synthetic lethality since *dna2–1* is not a mutator mutation and thus catastrophic mutagenesis is not likely to occur in the *dna2 pol3–01* mutant ([8]; unpublished data). OFP is the third process proposed to require pol δ exonuclease, since *pol3–01* is synthetically lethal with *rad27*Δ [81]. Mechanistically, this has been rationalized by the fact that pol δ 3′ exonuclease is an inhibitor of the strand displacement activity of pol δ during in vitro DNA synthesis [33]. In the absence of its 3′ exonuclease, pol δ can no longer idle at a nick, since the 3′ displaced flaps that form as intermediates in idling cannot be removed nucleolytically. Instead, pol δ processively displaces the strand having a 5′ terminus [33]. This is consistent with the idea of *dna2 pol3–01* lethality stemming from the failure to efficiently process excessively long 5′ flaps on Okazaki fragments. *pol δ-DV* is another exonuclease-deficient allele of pol δ, and *rad27-d* is a proliferating cell nuclease antigen noninteracting mutant of *rad27*. Overproduction of Dna2p suppresses the lethality of a *pol3 d-DV rad27-p rad51*Δ strain [11], supporting our conclusions.

*dna2–1* and *dna2–2* mutants are suppressed by deleting the nonessential *POL32*-encoded subunit of pol δ that is required for optimum strand displacement. Pol32p is required for efficient in vitro DNA replication by pol δ in the presence of replication factor C, proliferating cell nuclease antigen, and a primed template. The ability to displace 5′ ends is drastically decreased for pol δ lacking Pol32p [35,43], and the pol δ complex is expected to be defective in strand displacement synthesis in *pol32*Δ strains, thus reducing the need for Dna2p. Pol32p has also been shown to interact with pol α, and the same mutant that is synthetically lethal with *dna2–2*, *pol1–1* (G493R), is synthetically sick with *pol32*Δ [82]. This might hint at coordination between Okazaki fragment initiation and elongation, although there is no reported phenotype associated with a mutation *(pol32–8)* that disrupts the Pol32p–pol α interaction. While this manuscript was being prepared, it was reported that a mutation in *cdc27* ^+^, the *Schizosaccharomyces pombe* ortholog of *POL32,* suppresses one allele of *S. pombe dna2* [83], so this suppression is conserved. Why is *pol32*Δ r*ad27*Δ lethal [18] while *pol32*Δ *dna2* grows more robustly than *dna2* mutants? Loss of *pol32*Δ may shift the course of OFP from a flap removal pathway to one employing RNaseH. In the RNaseH pathway, FEN1 exonuclease may become essential to remove the last ribonucleotide, an activity that Dna2p does not appear to possess.

#### Interaction with Pol α and Primase (and Mcm10p).

Our demonstration here of synthetic lethal interactions of *dna2* with *pol1–1* and with *pri1-M4,* components of pol α–primase, may also fortify the argument that Dna2p participates in OFP. Although *pri1-m4* has an S phase checkpoint defect in addition to a DNA replication defect, the replication defect is more likely to be responsible for the synthetic lethality with *dna2,* given our data that *dna2* is not synthetically lethal with any of the mutations in major checkpoint genes, including *mec1*Δ *sml1*Δ. Certain alleles of *dna2* are also synthetically lethal with *mcm10–1* [23]. Recently, *MCM10* has been implicated in elongation and in stabilizing pol α in vivo as well as in stimulating pol α in vitro [84,85]. The interdependent functions of *DNA2* and *MCM10* may reflect an interaction in lagging-strand replication. Alternatively, Dna2p might play a role in repair of *mcm10–1*-generated damage. The combined new data on interactions between Dna2p, pol Δ, pol α, and primase may be evidence for a previously unexpected coupling of primer synthesis, polymerase switching, and primer removal.

### Differences between the Genetic Interactions of *DNA2* and Those of *RAD27*

Comparison of the data presented here (see Figure 2; Table S2) and the results of a similar thoroughly validated SGA screen using *rad27*Δ as a query gene [86] reveals a wide (and unanticipated) divergence between genes that are synthetically lethal with *dna2* and those that are synthetically lethal with *rad27*Δ. This divergence implies that the two enzymes may have slightly different sets of substrates. As pointed out in Results, the synthetic lethality of *rad27*Δ [69,87], but not *dna2,* with checkpoint mutants and with recombination mutants suggests that *rad27*Δ mutants probably accumulate more single-stranded DNA (the proposed signal for checkpoint activation) and more DSBs (repaired by recombination) [87], than *dna2* mutants do. If *dna2* mutants accumulate less damage than *rad27*Δ mutants, this in turn might suggest that FEN1 is the major OFP nuclease and that Dna2p is required at fewer (or different) sites than FEN1 [88]. This conclusion is consistent with other previously published evidence (despite the potential conundrum that deleting *DNA2* is lethal and deleting *RAD27* is not). First, pol δ, proliferating cell nuclease antigen, and FEN1 appear to act in a highly concerted fashion on templates that are optimal for pol δ efficiency in vitro, with little evidence that flaps longer than a few nucleotides are ever produced [10,33]. Second, *dna2* mutants are weak mutators, while *rad27*Δ mutants are strong mutators, as measured by point mutations or stability of di- or trinucleotide repeats, or even larger repeats [16,87,89–91]. Dna2p may be specialized to function in OFP in genomic locations where the DNA sequence poses problems for pol δ, creating flaps that are not good substrates for FEN1. These regions are likely to include the rDNA and telomeres, since we have shown significant replication defects in these loci in *dna2* mutants [17,35–37]. The role of Dna2p is not likely to be limited to these regions, however, as our previous immunofluorescence and chromatin immunoprecipitation analyses show Dna2p to be located at many other genomic regions during S phase [17]. Possible sites are replication slow zones or the inverted Ty repeats that give rise to genomic instability.

If FEN1 is the major flap nuclease, either Dna2p might help FEN1 on some flaps or Dna2p might recognize discrete subsets of flaps and process them independently. The genetic differences observed, combined with the quantitative biochemical data that show that Dna2p is very inefficient at stimulating FEN1, even on long flaps, direct future attention to potentially independent roles for Dna2p and FEN1.

### The Helicase Network for Preserving Genomic Stability

Previous screening of the nonessential gene knock-out collection with *sgs1*Δ and *srs2*Δ as queries identified a so-called helicase network defined by a set of common interactions [92]. Genes in this network are implicated by dozens of recent studies in the repair of damaged replication forks through sister chromatid recombination and replication restart mechanisms, but coupling of the network to DNA replication remains poorly understood (e.g., [93]). By reversing the screening process and using an essential lagging-strand replication gene as query, we have found that *dna2* is synthetically lethal with mutations in all of these helicases and in the genes with which they interact. The interactions shown in Figures 1 and 2 and our subsequent work (see Table 2) suggest that Dna2p may be one of the major replication proteins that coordinate this helicase network and replication.

The synthetic lethality of *dna2–2 sgs1*Δ and its lack of suppression by *rad51*Δ (Table 2) suggests that Sgs1p participates directly in DNA replication by aiding Dna2p in stimulating flap cleavage during OFP under some circumstances. This interpretation is attractive since the human Sgs1p orthologs, BLM and WRN, interact physically with Dna2p and suppress the replication defects in *dna2–1* mutants when overexpressed in yeast [94,95]. (The suppression could not be investigated with yeast *SGS1,* since its overproduction is toxic [A. Morgan, personal communication; unpublished data].) The reproducible effect of deleting *RAD51* in restoring defective growth to *dna2–2 sgs1*Δ mutants, however, is consistent with *SGS1* playing an additional role in a late stage of recombinational repair of *dna2*-induced lesions, such as in the resolution of Holliday junction intermediates into viable products, a reaction that requires Sgs1p in conjunction with Top3p [96]. Dna2p might be required to remove 5′ ends of nascent DNA in reversed forks, while Sgs1p serves as a helicase to resolve the forks, like RecJ and RecQ in bacteria [97]. Lesions due to *DNA2* deficiency do have the potential to lead to lethal recombination intermediates, because we find that *dna2* is synthetically lethal with *srs2*Δ and that this lethality is efficiently suppressed by deleting *RAD51*. *SRS2,* given its role in inhibiting an early step in recombination [98–100], probably prevents *dna2–2 sgs1*Δ–derived lesions from entering the recombinational repair pathway. Thus, the interaction between Sgs1p and Dna2p is multipotential.

Rrm3p promotes DNA replication through non-nucleosomal protein–DNA complexes, including the rDNA RFB, Rap1p-binding sites in the telomere, inactive ARS sites, and centromere DNA [101–104]. Rrm3p may act on the same replication intermediates as Dna2p rather than on downstream toxic intermediates formed during repair of faulty replication, since *rad51* mutations do not suppress the *dna2–2 rrm3*Δ synthetic lethality. Recently, *rrm3*Δ has been tested for synthetic lethality against a number of candidate gene deletions. Unlike *dna2–2 rrm3*Δ, most of the synthetically lethal combinations, such as *rrm3*Δ *srs2*Δ and *rrm3*Δ *sgs1*Δ, were suppressed by recombination mutants [47,75]. Thus, early replication intermediates cause cell lethality in *dna2 rrm3*Δ mutants, while recombination intermediates cause cell lethality in *rrm3*Δ *sgs1*Δ mutants. These intermediates may not involve FEN1, since *rad27*Δ *rrm3*Δ is not synthetically lethal [47]. Although both *dna2* and *rrm3*Δ mutants show significant pausing in the rDNA, a *fob1*Δ mutation did not restore viability to the *dna2–2 rrm3*Δ mutant (Table 2), so there are additional sequences replicated by these genes. We have recently demonstrated that Dna2p can stimulate FEN1 cleavage of long flaps with secondary structure, but that the reaction is inefficient [12,105]. Since Rrm3p is a 5′ to 3′ helicase, Rrm3p is a candidate for a helicase that may aid Dna2p in flap processing.

### DNA Repair

It does not appear that *dna2* interacts with genes involved in nucleotide excision repair, consistent with the relative resistance of *dna2* mutants to UV irradiation [16]. Base excision repair (long patch) involves all of the proteins also involved in OFP, and therefore a role for Dna2p in base excision repair would be supported by the interactions found in this work and the MMS sensitivity of *dna2* mutants [8]. A role for Dna2p in an unidentified Rad52p-dependent pathway was discussed above.

The synthetic lethality of *dna2* with each member of the Mre11p complex may contribute to emerging evidence that the Mre11p complex functions at the replication fork. First, the seven genes whose interactions overlapped most significantly with *dna2* (see Figure 2) are also synthetically lethal with the genes of the Mre11p complex. Second, the Mre11p complex associates with chromatin primarily during S phase, and this association does not appear to require DSBs [106]. It has been suggested that the Mre11p complex assists sister chromatid association [106], and that this association is required for recombinational repair of DSBs during DNA replication. Another view derives from the fact that the Mre11p complex and Exo1p are both required for activation of the Rad53p checkpoint kinase after inhibition of replication by HU. This leads to the inference that the Mre11p complex and Exo1p may convert DSBs arising at stalled replication forks into single-stranded DNA, a signal for subsequent repair. Since replication forks stall in *dna2* mutants, the synthetic lethality with mutations in Mre11p complex components or Exo1p could be explained by a failure to produce the single-stranded DNA signal. It is possible that DSBs that arise during normal DNA replication are repaired in Mre11p complex mutants, but are lethal in cells lacking both Mre11p and Dna2p [7]. Finally, or alternatively, the synthetic lethality of *dna2 mre11*Δ may indicate that *DNA2* is involved in a telomere defect, as has been shown in *S. pombe* [48,107]. We cannot eliminate the possibility that the Dna2p/Mre11p interaction is involved in repair, but we note that *dna2–2* and *rad50–5,* which is as deficient in repair as *rad52*Δ, are epistatic with respect to repair.

### Sister Chromatid Cohesion

Replication forks in *dna2* mutants pause at the RFB in the rDNA, where DSBs result [17,35,37]. Our current finding that deletion of *FOB1* suppresses the synthetic lethality of *dna2–2 ctf4*Δ damage could be explained if Ctf4p-mediated sister chromatid cohesion is necessary to repair damage at the RFB due to defective Dna2p. This requirement for cohesion in *DNA2* mutants is not limited to the rDNA, since the *dna2–2 ctf4*Δ *fob1*Δ strain is ts and radiation sensitive. Thus, the Dna2p deficiency must give rise to damage requiring cohesion for repair elsewhere in the chromosome as well. A role for cohesion in maintaining the replication fork during stalling or collapse is attractive since cohesion is required for efficient DSB repair [108]; a role for sister chromatid cohesion in preventing excessive sister chromatid exchange due to breaks at the RFB has been directly demonstrated [109]. We note that our analysis of replication in *dna2–2* strains by two-dimensional gel electrophoresis indicates a high incidence of stalled replication forks at sequences throughout the rDNA, not limited to the RFB, suggesting general replication fork stalling in *dna2* mutants and providing physical evidence of a more delocalized requirement for sister chromatid cohesion, perhaps throughout the chromosome [35,37].

### Chromatin Remodeling, Disassembly, and Reassembly

During DNA replication, the chromatin in front of the replication fork is disassembled and then reassembled behind the fork. Our new findings add to recent findings from many sources that are providing the first insight into the molecular links between the replication machinery and chromatin dynamics. Dna2p interacts with both Asf1p and yFACT. The Asf1p/Dna2p interaction in chromatin assembly/remodeling is too ill-defined for further inferences at the moment. However, the allele-specific synthetic lethality between *dna2* and *pol1–1* suggests that Dna2p participates in the recently demonstrated interplay between Spt16p (a component of the FACT-like nucleosome reorganization factor), Ctf4p, and pol α [56]. The *pol1–1* mutant protein fails to interact with Spt16p and shows altered temporal interaction with Ctf4p. The compromised association between Spt16p and pol α in the *pol1–1* mutant is accompanied by a delay both in pol α recruitment to late origins and its release, leading to a slow S phase [56]. By adding a link between Dna2p and this particular aspect of pol α function, our results support the model of the Formosa lab that the yeast Spt16p complex is likely to be directly involved in DNA replication [52–54,110], as has also been suggested for the frog FACT complex [111]. The Spt16p remodeling complex may facilitate the movement of pol α and Dna2p through nucleosomes, as proposed for human FACT in transcription [112,113]. The caveat that yFACT defects might result in reduced transcription of gene(s) that interact with *DNA2* was mentioned above.

Other *DNA2*-interacting genes encode specific sets of histone modification enzymes that catalyze histone ubiquitylation, methylation, and deacetylation. We discovered here that the synthetic sickness of *dna2* and *rad6* is related to Rad6p/Bre1p-mediated ubiquitylation of histone H2B, which in turn leads to methylation of H3 at the lysine at position 4 by the SET1 complex, containing Set1p, Swd1p, and Swd3p [114]. *set1* mutants are sensitive to HU and may accumulate DNA damage during S phase. *set1* was not recovered in the SGA screen because it is not in the deletion collection. Ubiquitylation and methylation alter silencing and chromatin structure at the rDNA and at telomeres, which may suggest a mechanism for interaction with *dna2* [114,115].

The interaction between Dna2p and Hst3p and the Rpd3p/Pho23p histone deacetylases is interesting because mutations in ORC, the replication initiator, as well as *rad27*Δ, *pol32*Δ, and s*gs1*Δ also show synthetic interaction with *hst3*Δ [18,116]. *HST3* performs a redundant function in DNA replication but *hst3 hst4* cells have phenotypes indicative of replication defects, such as increased rates of chromosome loss and mitotic recombination, decreased telomere silencing, and hypersensitivity to UV treatment [117]. *DNA2* is the only DNA replication gene thus far found to interact with *RPD3*. Histone deacetylation by the Rpd3p/Pho23p complex has been previously implicated in the temporal regulation of origin activation, but not elongation, in DNA replication [67,68]. The observation that *hst1*Δ, *hst3*Δ, *rpd3*Δ, *bre1*Δ, *spt16*Δ, *cac2*Δ, and *vid21*Δ are all linked to the network shown in Figure 2 suggests that the existing nucleosome structure has been optimized for high fidelity DNA replication (see Protocol S1).

### Conclusions

We propose that the genome maintenance network is coordinated by physical interaction of the replication proteins with the complexes that carry out regulation and repair. A good deal of evidence supports this. Originally we found that Dna2p co-purified with FEN1 and later demonstrated the genetic interaction. We discovered a genetic interaction between Dna2p and BLM and WRN helicases—the human orthologs of Sgs1p—and then found that this genetic interaction also represented a direct physical interaction between Dna2p and those helicases [94]. Others found genetic interactions between *DNA2* and *RFA1,* encoding the single-stranded DNA binding protein RPA, and similarly documented a physical interaction between the proteins Dna2p and RPA [13,118]. Indeed, most DNA replication proteins multitask and are components of many complexes. Pol δ and ɛ are involved in DNA repair complexes as well as replication complexes. Pol ɛ is, in addition, involved in the S phase checkpoint. FEN1 itself is involved in multiple reactions, including long patch base excision repair [15]. Though circumstantial, the resemblance between the basic topology of the genetic network described here and the proteomic network described in bacteria [76] further suggests the model that replication proteins physically coordinate repair and regulatory genome maintenance complexes. We anticipate that this model will be verified in detail when proteomic approaches in yeast yield the kind of comprehensive data that can already be obtained from genetic screens such as SGA.

## Materials and Methods

### Strains.

All strains used in the study are found in Table S4. The strains used in the SGA screen and subsequent verification were as follows: 4741 *MATa his3Δ1 leu2Δ0 met15Δ0 ura30Δ*; 4741 *Mat* α *his3Δ1 leu2Δ0 met15Δ0 ura3Δ0;* strain 4741dna2–1–6D (MB100) MATa *dna2-1his3*Δ*1 leu2*Δ*0 met15*Δ*0 ura3*Δ*0;* strain 4741dna2–2–11D (MB101) *MATα dna2–2::LEU2 his3*Δ*1 leu2*Δ*0 met15*Δ*0 ura3*Δ*0;* Y3656 *can1*Δ*::MFA1pr-HIS3-Mf*α*1pr-LEU2 his3*Δ*1 leu2*Δ*0 met15*Δ*0 ura3*Δ*0;* Y3656dna2–1; Y3656 *dna2–1::URA3;* Y3656dna2–2; Y3556 *dna2–2::URA3;* and W30W303 *MATa ade2–1 can1–100 his3–11,15 leu2–3,112, trp1–1 ura3 RAD5*. Y3656 is derived from strain 4741 as described [19]. Unless otherwise noted, all haploid deletion mutants used in this work were in strain 4741 and all double mutants were tested using 4741dna2–1 or Y3656dna2–2 as indicated.

### SGA screen.

The SGA screen was performed as previously described [19]. Y3656dna2–1 and Y356dna2–2 were constructed for this study and used as query strains. SGA analysis was performed for each of the *dna2* alleles. Genes that showed synthetic lethality or synthetic sickness in the primary screen were tested by standard tetrad dissection [119]. For the secondary tetrad analysis, new heterozygous diploids were constructed between MB100 (4741 *dna2–1*) and MB101 (4741 *dna2–2*) and each of the candidate deletion mutants in strain 4741 (Invitrogen, Carlsbad, California, United States). Thus, each synthetic interaction that is reported here was tested using two independent diploids, one in the original screen and one in the secondary screen. The *dna2–2* dissections were incubated at 30 °C and *dna2–1* dissections at 23 °C. Any strain failing to generate viable double mutants was deemed synthetically lethal. A strain was considered synthetically sick if tetrads gave fewer double mutants than expected and double mutants grew slower than either single mutant. At least ten tetrads were dissected for each double mutant, and usually more were dissected.

### X-ray and MMS sensitivity.

The X-ray source was Pantak (East Haven, Connecticut, United States) MK II 70 kev 20 ma. The source was calibrated and experiments were carried out as previously described [16]. For the MMS sensitivity assay, 0.005% and 0.01% MMS was added to yeast-peptone-dextrose (YPD) plates after autoclaving, and plates were used the same day.

## Supporting Information

Protocol S1 Other (53 KB DOC)

Table S1 Genes That Show Little, If Any, Interaction with *dna2–1* or *dna2–2* in Secondary Screen (107 KB PDF)

Table S2 Synthetic Lethal Interactions Used to Prepare Figure 2
(111 KB PDF)

Table S3 Number of Spores after Dissection of *dna2–2 mec1 tel1 slm1* Heterozygote (95 KB PDF)

Table S4 Strains Used (103 KB PDF)

## Abbreviations

DSB: double-strand break
FAA: 2-amino-5-fluorobenzoic acid
HU: hydroxyurea
MMS: methyl methane sulfonate
OFP: Okazaki fragment processing
pol: DNA polymerase
RFB: replication fork barrier
SGA: synthetic genetic array
ts: temperature-sensitive
WT: wild-type
YPD: yeast-peptone-dextrose
