# Supplementary material for: A Network of Multi-Tasking Proteins at the DNA Replication Fork Preserves Genome Stability
Source: PLoS Genet. 2005 Dec 2;1(6):e61. doi: 10.1371/journal.pgen.0010061 (PMC1298934; doi:10.1371/journal.pgen.0010061)
Supplement: Supplementary file 1 [file pgen.0010061.st001.pdf]

Supplementary Information

Table S1. Genes that Show Little, if any, Interaction with *dna2-1* or *dna2*-2 in Secondary Screen.

dna2-1	*dna2-2*
atp18	*atp18*
bre1	*cbp1*
caf20	*ces3*
cbp1	*chl1*
ces3	*clb2*
chb1	*esc2*
cls2	*gyp6*
csm1	*hir3*
dcr1	*hog1*
esc1	*hst3*
hir3	*ioc2*
ilv6	*ira2*
ira2	*kin2*
kar3	*lge1*
kin2	*mrp49*
lge1	*mum2*
mum2	*nit1*
nit1	*pak3*
phb1	*phb1*
prs5	*prs5*
psk3	*ptc2*
ptc2	*rad10*
rad10	*rad18*
rad18	*rad4*
rad4	*rad54*
rad54	*rad55*
rad55	*rnh202*
rnh203	*rnh203*
sap30	*rnh35*
slx5	*sin3*
swd1	*sum1*
swi4	*swd1*
top3	*swd3*
vga3	*swi4*
yen1	*ubd6*
	ygr081c
