# Supplementary material for: A Network of Multi-Tasking Proteins at the DNA Replication Fork Preserves Genome Stability
Source: PLoS Genet. 2005 Dec 2;1(6):e61. doi: 10.1371/journal.pgen.0010061 (PMC1298934; doi:10.1371/journal.pgen.0010061)
Supplement: Supplementary file 2 [file pgen.0010061.st002.pdf]

Table S2. Synthetic Lethal Interactions Used to Prepare Figure 1B.

	DNA2	*RAD27*	*SGS*	*SRS2*	*RRM3*	*POL32*
DNA2		SL	SL	SL	SL	**SR**
SGS1	SL	SL		SL	SL	
SRS2	SL	SL	SL		SL	SL
RRM3	SL		SL	SL		
NUCLEASES INVOLVED IN OKAZAKI FRAGMENT PROCESSING						
RAD27	SL		SL	SL		SL
EXO1	SL	SL				
RNH35	SL	SS	SS			
RNH202	SL	SS	SS			
RNH203			SS			
YEN1	SS					
TOPOISOMERASES						
TOP3	SS			SL	SL	
TOP1			SL			
rDNA						
FOB1	**SR**		**SR**			
DNA REPLICATION						
POL32	**SR**	SL	SL	SL		
POL3-01	SL	SL				
POL1	SS					
RPA1	SL					
MGS1			SL			
CDC8		SL	SL			
RNR1		SL				
CDC7			SL			
MCM10	SL					
CDC45		SL			SL	SL
ELG1	SL	SL	SL	SL	SL	SL
DNA REPAIR						
RAD50	SL	SL	SL	SL	SL	SL
XRS2	SL	SL	SL	SL	SL	SL
MRE11	SL	SL	SL	SL	SL	SL
RAD51		SL				SL
RAD52	SS	SL		SL	SL	SL
RAD54		SL		SL		SL
RAD55		SL				SL
SAE2	SL	SL	SL		SL	
MMS2				SL		
RAD4		SL				
MMS1	SL			SL	SL	

MMS22	SL			SL	SL	
WSS1			SL			

HYDROXYUREA ALTERED SENSITVITY

HUR1					SL	
SHU2				SL		
RMD7						

CELL CYCLE

SWE1			SL			
HPC2		SL				
CLA4	SS					
LTE1						SL
PPH3					SL	

CELL CYCLE CHECKPOINTS

MEC1		SL				
DUN1						SL
RAD9	**SR**	SL			SL	SL
RAD17		SL				SL
DDC1		SL		SL		SL
RAD24		SL				SL
TOF1	SL	SL				SL
MRC1	SL	SL	SL	SL	SL	SL
CSM3	SL	SL	SL	SL		SL

TELOMERES

PIF1	**SR**				**SR**	
TEL1	SS(37)					
EST1	SS					
EST2	SS					

CHROMOSOME COHESION

CTF4	SL	SL	SL	SL	SL	SL
CHL12	SL	SL	SL	SL		SL
CHL1		SL		SL		
CTF8					SL	
DCC1		SL	SL	SL	SL	SL

SLX GENES

MUS4		SL	SL			
MMS81		SL	SL			
SLX4			SL			
SLX5	SL		SL			
SLX8	SL		SL			
SL9			SL			
SLX1			SL			

CHROMATIN REMODELING AND SILENCING PROTEINS

RAD6	SS					
BRE1	SS					
SWD1	SS					

SWD3	SS		SL			
SAP30	SS					
LGE1	SS					
RTF1	SS					
ASF1	SS		SL			
HST1		SL				
HST3	SL	SL	SL			SL
RPD3	SL					
PHO23	SL					
ESC2		SL	SL	SL	SL	SL
ESC4			SL	SL	SL	
RTT103	SS					
NCE4				SL	SL	SL
CAC2		SL				
CAC3						SL
RTT101					SL	

UBIQUITIN AND SUMO METABOLISM AND PROTEIN DEGRADATION

UBC4	SS					
SMT3				SL		
RAD6	SL					

STRESS

HOG1	SL					
SOD1	SL	SL	SL			
SOD2					SL	
LYS7	SL	SL				
HAL3			SL			
HSP40						SL
SIS2		SL	SL			

MITOCHONDRIA

GDS1				SL		
YPR116W		SL				

RNA INTERACTING PROTEINS

CAF20	SL					
MSL1				SL		
MPH1				SL		
Pub1			SL			

PROTEIN SYNTHESIS

RPL27A		SL				
RPS30B		SL				
RPL24A			SL			

MISMATCH REPAIR

MSH6			SL			
PRM1					SL	

CELL POLARITY AND MORPHOLOGY

MNN10						SL
HOC1						SL
MEIOSIS						
EAF1						SL
VACUOLAR SORTING						
VMA6		SL				

SL-Synthetically lethal
SS-Synthetic sick
SR-Synthetic rescue, enhanced growth of double mutant
Data in this table are compiled from this study, and from references (Tong et al., 2001; Ooi et al., 2003; Tong et al., 2004; Torres et al., 2004, Nicolas, in press).
