# Supplementary material for: A Network of Multi-Tasking Proteins at the DNA Replication Fork Preserves Genome Stability
Source: PLoS Genet. 2005 Dec 2;1(6):e61. doi: 10.1371/journal.pgen.0010061 (PMC1298934; doi:10.1371/journal.pgen.0010061)
Supplement: Supplementary file 3 [file pgen.0010061.st003.pdf]

Table S3. Number of Spores after Dissection of *dna2-2 mec1 tel1 slm1* Heterozygote.

Genotype				Spores
dna2-2	*mec1*	*tel1*	*sml1*	
WT	*WT*	*WT*	*WT*	4
dna2-2				11
	mec1			1
		tel1		11
			sml1	7
dna2-2	*mec1*			0
dna2-2		*tel1*		7
dna2-2			*sml1*	4
	mec1		*sml1*	8
		tel1	*sml1*	7
dna2-2	*mec1*		*sml1*	12
dna2-2		*tel1*	*sml1*	5
	mec1	*tel1*	*sml1*	6
dna2-2	*mec1*	*tel1*	*sml1*	9
