# Supplementary material for: A Network of Multi-Tasking Proteins at the DNA Replication Fork Preserves Genome Stability
Source: PLoS Genet. 2005 Dec 2;1(6):e61. doi: 10.1371/journal.pgen.0010061 (PMC1298934; doi:10.1371/journal.pgen.0010061)
Supplement: Supplementary file 4 [file pgen.0010061.st004.pdf]

Table S4. Strains Used.

Strain	Genotype	Source
4741	*MATa his3Δ1 leu2Δ0 met15Δ0 ura3Δ0*	IN-VITROGEN
4742	*MATα his3Δ1 leu2Δ0 met15Δ0 ura3Δ0*	IN-VITROGEN
Y3656	*can1Δ::MFA1pr-HIS3-Mha1pr-LEU2 his3Δ1 leu2Δ0 met15Δ0 ura3Δ0*	(Tong et al., 2001)
579-6D (W303)	*MATa trp1-1 ura3-1 his3-11,15 leu2-3,112 ade2-1 can1-100 RAD5*	H.Klein
580-10D (W303)	*MATα trp1-1 ura3-1 his3-11,15 leu2-3,112 ade2-1 can1-100 RAD5*	H.Klein
W303dna2-1	*MATa dna2-1 trp1-1 ura3-1 his3-11,15 leu2-3,112 ade2-1 can1-100 RAD5*	(Weitao et al., 2003a; Weitao et al., 2003b)
YLL157	*MATa rad9Δ::URA3 trp1-1 ura3-1 his3-11,15 leu2-3,112 ade2-1 can1-100 RAD5*	(Longhese et al., 1997)
SPY40	*MATa tel1::URA3 trp1-1 ura3-1 his3-11,15 leu2-3,112 ade2-1 can1-100*	(Ritchie et al., 1999)
U953-61A	*MATa mec1Δ::TRP1 sml1::HIS3 trp1-1 ura3-1 his3-11,15 leu2-3,112 ade2-1 can1-100*	(Zhao et al., 1998)
YAO328	*MATa mrc1AQ::LEU2::mrc1Δ::URA3 trp1-1 ura3-1 his3-11,15 leu2-3,112 ade2-1 can1-100*	Steve Elledge
Y2298	*MATa HIS3::mrc1AQ MYC13 trp1-1 ura3-1 his3-11,15 leu2-3,112 ade2-1 can1-100*	(Osborn and Elledge, 2003)
MB100	*MATa dna2-1 his3Δ1 leu2Δ0 met15Δ0 ura3Δ0*	This study
MB101	*MATα dna2-2-LEU2 his3Δ1 leu2Δ0 met15Δ0 ura3Δ0*	This study
MB103	*can1Δ::MFA1pr-HIS3-Mha1pr-LEU2 dna2-1::URA3 his3Δ1 leu2Δ0met15Δ0 ura3Δ0*	This study
MB104	*can1Δ::MFA1pr-HIS3-Mha1pr-LEU2 dna2-2URA3 his3Δ1 leu2Δ0met15Δ0 ura3Δ0*	This study
MB110	*MATa dna2Δ::kanMX his3Δ1 leu2Δ0 met15Δ0 ura3Δ0(pSEY18GALDNA2)*	This study
MB111	*MATa dna2ΔkanMX pif1Δ::HIS5 his3Δ1 leu2Δ0 met15Δ0 ura3Δ0*	This study
MB112	*MATα dna2-2-LEU2 pif1Δ::kanMX his3Δ1 leu2Δ0 met15Δ0 ura3Δ0*	This study
509	*MAT□ pif1Δ::kanMX his3Δ1 leu2Δ0 met15Δ0 ura3Δ0*	IN-VITROGEN
MB90-7A	*MATa dna2-1 trp1-1 ura3-1 his3-11,15 leu2-3,112 ade2-1 can1-100*	This study
MB91	*MATa dna2-1pif1Δ::HIS5 trp1-1 ura3-1 his3-11,15 leu2-3,112 ade2-1 can1-100*	This study
24D	*MATα fob1Δ::HIS3 his3Δ1 leu2Δ0 met15Δ0 ura3Δ0*	This study
27A	*MATa ctf4Δ::kanMX fob1Δ::HIS3 his3Δ1 leu2Δ0 met15Δ0 ura3Δ0*	This study
2D	*MATα dna2-2::LEU2 fob1Δ::HIS3 his3Δ1 leu2Δ0 met15Δ0*	This study

	ura3Δ0	
25A	*MATα dna2-2::LEU2 ctf4Δ::kanMX fob1Δ::HIS3 his3Δ1 leu2Δ0 met15Δ0 ura3Δ0*	This study
4A	*MATa dna2-2::LEU2 pol32Δ::kanMX his3Δ1 leu2Δ0 met15Δ0 ura3Δ0*	This study
2D	*MATα dna2-1 pol32Δ:: kanMX his3Δ1 leu2Δ0 met15Δ0 ura3Δ0*	This study
17B	*MATa dna2-2:LEU2 sml1Δ::HIS3 trp1-1 ura3-1 his3-11,15 leu2-3,112 ade2-1 can1-100*	This study
31A	*MATα mec1Δ::TRP1 tel1Δ::URA3 sml1Δ::HIS3 trp1-1 ura3-1 his3-11,15 leu2-3,112 ade2-1 can1-100*	This study
31C	*MATa dna2-2 mec1Δ::TRP1 sml1Δ::HIS3 trp1-1 ura3-1 his3-11,15 leu2-3,112 ade2-1 can1-100*	This study
23C	*MATa sml1Δ::HIS3 trp1-1 ura3-1 his3-11,15 leu2-3,112 ade2-1 can1-100*	This study
37C	*MATα tel1Δ::URA3 sml1Δ::HIS3 trp1-1 ura3-1 his3-11,15 leu2-3,112 ade2-1 can1-100*	This study
36D	*MATα mec1Δ::TRP1 sml1Δ trp1-1 ura3-1 his3-11,15 leu2-3,112 ade2-1 can1-100*	This study
35A	*MATα dna2-2::LEU2 tel1Δ::TRP1 sml1Δ::HIS3 trp1-1 ura3-1 his3-11,15 leu2-3,112 ade2-1 can1-100*	This study
	pol1-1	(Lucchini et al., 1990)
CY387	*pri1-M4*	(Marini et al., 1997)
