# Supplementary material for: A Network of Multi-Tasking Proteins at the DNA Replication Fork Preserves Genome Stability
Source: PLoS Genet. 2005 Dec 2;1(6):e61. doi: 10.1371/journal.pgen.0010061 (PMC1298934; doi:10.1371/journal.pgen.0010061)
Supplement: Supplementary file 5 [file pgen.0010061.sd001.doc]

### Protocol S1.

Yen1: *Yen1* mutants are also synthetically sick with deletions of *YBR094w*, a member of the tubulin tyrosine ligase family. *YBR094w* might be involved in microtubule function, but the deletion mutation has no observable phenotype. A deletion of the *YBR094w* was found to be synthetically lethal with *cdc2-1, elg1, esc2, nce4, sgs1, top3,* and *tsa1*, and synthetically sick with *asf1, csm1, isc1, lys7, rrp6, soh1* in a SGA screen (Siede et al., 1989; Tong et al., 2001; Tong et al., 2004). This synthetic lethal interaction pattern links *YBR094w* to DNA replication and indirectly links Yen1 to DNA replication.

**Histone modification**

We have carried out several pairwise crosses with additional genes to explore the cause of *dna2 rpd3* lethality. The Rpd3 complex is involved in silencing gene expression (Loewith et al., 2001), and interacts with Ume6 and Ume1 in this function (Kurdistani et al., 2002). We find, however, that *dna2-1* strains are not synthetically lethal with *ume6* strains (this work, not shown), so this may also not explain the *dna2 rpd3Δ* synthetic lethality. Furthermore, strains with two copies of *SIR2* in the chromosome are not synthetically lethal with *dna2-1* and presumably these strains have enhanced silencing (Mays Hoopes et al., 2002). *rpd3Δ* mutants suppress the checkpoint defects of *mec1* and *rad9* strains by a mechanism dependent on *mad2*, a mediator of the spindle checkpoint (Scott and Plon, 2003). Howeve*r, mad2* mutants do not suppress the *dna2-1 rpd3Δ* synthetic lethality (this work, data not shown), suggesting that the synthetic lethality is not caused by checkpoint mediated cell arrest.

**Oxidative Stress- *sod1Δ, lys7Δ***

*dna2* mutants are synthetically lethal with *sod1Δ, lys7Δ,* and *tsa1Δ.* Thus, Dna2 may be involved in the network in yeast that is responsible for avoiding deleterious outcomes of oxidative damage during DNA replication (Huang and Kolodner, 2005). These interactions may also be related to the observation that defects in DNA replication in *S. pombe* (*orp2*, *dfp1*, and *cdc18* mutants) appear to cause increases in ROS (reactive oxygen species) production when assayed with 2',7'-dichlorodihydrofluorescein (Burhans et al., 2003). Not only *dna2*, but also genes in the helicase network and *rad27* are also synthetically lethal with these genes (Ooi et al., 2003; Huang and Kolodner, 2005). Further studies of this observation are now warranted, but too little is known presently to explain the observations. We have also carried out some preliminary experiments to investigate the reason for *dna2 sod1* lethality. As noted previously (Ooi et al., 2003), absence of SOD results in elevated levels of superoxide anions, which can inactivate enzymes containing [4Fe-4S] clusters, such as aconitase, succinate dehydrogenase, and possibly ribonucleotide reductase (De Freitas et al., 2000). Addition of iron allowed *sod1* mutants to grow to higher cells density in glycerol containing media, presumably by allowing reactivation of the inactivated [4Fe-4S] clusters in the mitochondria (Schmidt and Kolodner, 2004). Also, addition iron suppressed the lysine auxotrophy of *sod1* mutants, probably by reactivating homoaconitase (De Freitas et al., 2000). However, addition of iron to YPD dissection media did not rescue the *dna2-2 sod1* synthetic lethality. Therefore either free iron does not reactivate superoxide inactivated ribonucleotide reductase, or ribonucleotide reductase is not the target in *sod1* mutants. Overproduction of *RNR1*, however, does not suppress the *dna2 sod1Δ* synthetic lethality (data not shown). Another possibility is that, as proposed in *E. coli*, the superoxide ion destroys the tyrosyl radical essential for ribonucleotide reductase activity. Evidence to support this mechanism, however, is available only in *E. coli*.

**Osmotic Stress Genes**

Synthetic lethality of *dna2* with *hog1Δ* suggests that the osmotic stress pathway is required for viability of *dna2* mutants. We also found that this pathway is highly induced in *dna2* mutants late in their life span, using microarray analysis (Lesur and Campbell, 2004). We and others have reported that *dna2* mutants could be at least partially suppressed by addition of osmotic stabilizing agents, such as sorbitol to the growth medium. Many replication mutants are synthetically lethal with genes in this pathway.

**RNA Metabolism**

*dna2* is synthetically sick with *rtt103Δ* and lethal with *trf4Δ*. These interactions suggest that Dna2 may perform a backup role to the primary enzymes involved in RNA degradation during mRNA processing (Kadaba et al., 2004; Kim et al.)

**References for Supplemental Material**

Burhans, W. C., Weinberger, M., Marchetti, M. A., Ramachandran, L., D'Urso, G., and Huberman, J. A. (2003). Apoptosis-like yeast cell death in response to DNA damage and replication defects. Mutat. Res. *532*, 277-243.

De Freitas, J. M., Liba, A., Meneghini, R., Valentine, J. S., and Grallla, E. B. (2000). Yeast lacking the Cu-Zn superoxide dismutase show altered iron homeostasis. J. Biol. Chem. *275*, 11645-11649.

Huang, M.-E., and Kolodner, R. D. (2005). A biological network in *Saccharomyces cerevisiae* prevents the deleterious effects of endogenous oxidative DNA damage. Mol. Cell *17*, 709-720.

Kadaba, S., Krueger, A., Trice, T., Krecic, A. M., Hinnebusch, A. G., and Anderson, J. (2004). Nuclear surveillance and degradation of hypomodified initiator tRNA^Met^ in *S. cerevisiae*. Genes Dev. *18*, 1227-1240.

Kim, M., Krogan, N. J., Vasiljeva, L., Rando, O. J., Nedea, E., Greenblatt, J. F., and Buratowski, S. (2004). The yeast Rat1 exonuclease promotes transcription termination by RNA polymerase II. Nature *432*, 517-522.

Kurdistani, S. K., Robyr, D., Tavazoie, S., and Grunstein, M. (2002). Genome-wide binding map of the histone deacetylase Rpd3 in yeast. Nature Genet. *31*, 248-254.

Lesur, I., and Campbell, J. L. (2004). The transcriptome of prematurely aging yeast cells is similar to that of telomerase deficient cells. Mol. Biol. Cell *15*, 1297-1312.

Loewith, R., Smith, J. S., Meijer, M., Williams, T. J., Bachman, N., Boeke, J. D., and Young, D. (2001). Pho23 is associated wit the Rpd3 histone deacetylase and is required for its normal function in regulation of gene expression and silencing in *Saccharomyces cerevisiae*. J. Biol. Chem. *276*.

Longhese, M. P., Paciotti, V., Fraschini, R., Zaccarini, R., Plevani, P., and Giovanni, L. (1997). The novel DNA damage checkpoint protein Ddc1p is phosphorylated periodically during the cell cycle and in response to DNA damage in budding yeast. EMBO J. *16*, 5216-5226.

Lucchini, G., Falconi, M. M., Pizzagalli, A., Aguilera, A., Klein, H. L., and Plevani, P. (1990). Nucleotide sequence and characterization of temperature-sensitive *pol1* mutants of *Saccharomyces cerevisiae*. Gene *90*, 99-104.

Marini, F., Paciotti, V., Lucchini, G., Plevani, P., Stern, D. F., and Foiani, M. (1997). A role for DNA primase in coupling DNA replication to DNA damage response. EMBO J. *16*, 639-650.

Mays Hoopes, L. L., Budd, M., Choe, W., Weitao, T., and Campbell, J. L. (2002). Mutations in DNA replication genes reduce yeast life span. Mol. Cell. Biol. *22*, 4136-4146.

Ooi, S. L., Shoemaker, D. D., and Boeke, J. D. (2003). DNA helicase gene interaction network defined using synthetic lethality analyzed by microarray. Nature Genet. *35*, 277-286.

Osborn, A. J., and Elledge, S. J. (2003). Mrc1 is a replication fork component whose phosphorylation in response to DNA replication stress activates Rad53. Genes Dev. *17*, 1755-1767.

Ritchie, K. B., Mallory, J. C., and Petes, T. D. (1999). Interactions of TLC1 (which encodes the RNA subunit of telomerase), TEL1, and MEC1 in regulating telomere length in the yeast *Saccharomyces cerevisiae*. Mol. Cell. Biol. *19*, 6065-6075.

Schmidt, K. H., and Kolodner, R. D. (2004). Requirement of Rrm3 helicase for repair of spontaneous DNA lesions in cells lacking Srs2 or Sgs1 helicase. Mol. Cell. Biol. *24*, 3213-3226.

Scott, K. L., and Plon, S. E. (2003). Loss of Sin3/Rpd3 histone deacetylase restores the DNA damage response in checkpoint-deficient strains of *Saccharomyces cerevisiae*. Mol. Cell. Biol. *23*, 4522-4531.

Siede, W., Robinson, G., Kalainov, D., Malley, T., and Friedberg, E. C. (1989). Regulation of the *RAD2* gene of *Saccharomyces cerevisiae*. Mol. Microbiol. *3*, 1697-1707.

Tong, A. H. Y., Evangelista, M., Parsons, A. B., Xu, H., Bader, G. D., Page, N., Robinson, M., Raghibizadeh, S., Hogue, C. W. V., Bussey, H.*, et al.* (2001). Systematic genetic analysis with ordered arrays of yeast deletion mutants. Science *294*, 2364-2368.

Tong, A. H. Y., Lesage, G., Bader, G. D., Ding, H., Xu, H., Xin, X., Young, J., Berriz, G. F., Brost, R. L., Chang, M.*, et al.* (2004). Global mapping of the yeast genetic interaction network. Science *303*, 808-813.

Torres, J. Z., Schnakenberg, S. L., and Zakian, V. A. (2004). *Saccharomyces cerevisiae* Rrm3p DNA helicase promotes genome integrity by preventing replication fork stalling: Viability of *rrm3* cells requires the intra-S-phase checkpoint and fork restart activities. Mol. Cell. Biol *24*, 3198-3212.

Weitao, T., Budd, M., and Campbell, J. L. (2003a). Evidence that yeast SGS1, DNA2, SRS2, and FOB1 interact to maintain rDNA stability. Mutation Res. *532*, 157-172.

Weitao, T., Budd, M., Mays Hoopes, L. L., and Campbell, J. L. (2003b). Dna2 helicase/nuclease causes replicative fork stalling and double-strand breaks in the ribosomal DNA of *Saccharomyces cerevisiae*. J. Biol. Chem. *278*, 22513-22522.

Zhao, X., Muller, E. G. D., and Rothstein, R. (1998). A suppressor of two essential checkpoint genes identifies a novel protein that negatively affects dNTP pools. Mol. Cell *2*, 329-340.
